# Exploring New Bioorthogonal Catalysts: Scaffold Diversity in Catalysis for Chemical Biology

**DOI:** 10.1002/advs.202404431

**Published:** 2025-02-07

**Authors:** Yan Zhang, Qizhen Huang, Fang Lei, Wanlong Qian, Chengfeng Zhang, Qi Wang, Chaoqun Liu, Haiwei Ji, Faming Wang

**Affiliations:** ^1^ School of Public Health Nantong University Nantong 226019 China; ^2^ Institute of Special Environmental Medicine Nantong University Nantong 226019 China; ^3^ School of Pharmacy Henan University Kaifeng 475004 China

**Keywords:** biomedical application, bioorthogonal catalysis, inorganic materials, organic materials, scaffold diversity

## Abstract

Bioorthogonal catalysis has revolutionized the field of chemical biology by enabling selective and controlled chemical transformations within living systems. Research has converged on the development of innovative catalyst scaffolds, seeking to broaden the scope of bioorthogonal reactions, boost their efficiency, and surpass the limitations of conventional catalysts. This review provides a comprehensive overview of the latest advancements in bioorthogonal catalyst research based on different scaffold materials. Through an in‐depth analysis of fabrication strategies and applications of bioorthogonal catalysts, this review discusses the design principles, mechanisms of action, and applications of these novel catalysts in chemical biology. Current challenges and future directions in exploring the scaffold diversity are also highlighted. The integration of diverse catalyst scaffolds offers exciting prospects for precise manipulation of biomolecules and the development of innovative therapeutic strategies in chemical biology. In addition, the review fills in the gaps in previous reviews, such as in fully summarizing the presented scaffold materials applied in bioorthogonal catalysts, emphasizing the potential impact on advancing bioorthogonal chemistry, and offering prospects for future development in this field.

## Graphical abstract

This review provides a comprehensive and timely overview of the recent advances in scaffolded bioorthogonal catalysts. It discusses the design principles, mechanisms of action, and distinct features of these novel catalysts. Besides, it also emphasizes the potential impact of these unique catalysts on advancing bioorthogonal chemistry and offers prospects for future development in this field.

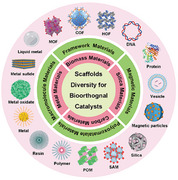

## 1 Introduction

Bioorthogonal chemistry is a rapidly emerging field that has enabled the development of novel chemical biology tools and applications.^[^ ^1^, ^2^, ^3^ ^]^ Within this field, bioorthogonal catalysis, which involves accelerating bioorthogonal reactions with catalysts, has gained widespread acceptance, as it expands the scope of bioorthogonal reactions in chemical biology, allowing for selective and controlled chemical transformations within living systems.^[^ ^4^, ^5^ ^]^ These bioorthogonal catalysts, have revolutionized our ability to manipulate and visualize biomolecules, unravel intricate biological processes, and develop innovative therapeutic strategies.^[^ ^6^, ^7^ ^]^ Conventional transition metal catalysts (TMCs) have been extensively employed for coupling reactions, cleavage reactions, and in‐situ synthesis‐related applications.^[^ ^8^, ^9^, ^10^ ^]^ At present, there's no denying that TMCs are still the main types of bioorthogonal catalysts.^[^ ^11^, ^12^ ^]^ For example, Pd has shown exceptional efficiency in Suzuki–Miyaura Coupling reactions,^[^ ^13^ ^]^ while Cu has demonstrated remarkable promise in click reactions.^[^ ^14^ ^]^ Furthermore, Au, Pd, and Pt have exhibited excellent catalytic activity in depropargylation reactions,^[^ ^15^, ^16^, ^17^ ^]^ and Ru and Ir have displayed significant potential in reduction reactions.^[^ ^18^, ^19^ ^]^ While they exhibit high catalytic activity and broad substrate scope, these catalysts often suffer from inherent disadvantages that limit their direct utility in biological systems.^[^ ^20^, ^21^, ^22^ ^]^ One major drawback is the potential cytotoxicity and adverse effects associated with the presence of transition metals.^[^ ^23^ ^]^ The introduction of exogenous transition metals into living organisms can disrupt cellular processes and compromise cell viability. Furthermore, metal catalysts can induce undesired side reactions, leading to non‐specific labeling, increased background noise, and compromised biological functionality.^[^ ^24^ ^]^ These limitations necessitate the development of alternative approaches to improve the biocompatibility and performance of bioorthogonal TMCs.

The catalysts based on scaffolded transition metals offer an innovative strategy to address the disadvantages. By utilizing specific scaffold designs, these catalysts provide distinct advantages that enhance their application in chemical biology.^[^ ^25^ ^]^ One notable advantage is the improved biocompatibility conferred by the scaffold, mitigating concerns of cytotoxicity and metal‐induced cellular perturbations. The scaffolding catalysts can shield the reactive transition metal center, reducing its exposure to biological components and minimizing off‐target effects. Additionally, The scaffolding TMCs can offer multifunctionality and synergistic effects.^[^ ^26^ ^]^ By integrating additional functional moieties within the scaffold, such as targeting ligands or imaging probes, these catalysts can simultaneously facilitate bioorthogonal reactions and impart additional biological functionalities. This multifunctionality enhances the versatility of the catalysts, enabling simultaneous labeling, imaging, or therapeutic interventions within the same biological system. The scaffolding catalysts can be further integrated with supramolecular chemistry or nanomaterials, providing opportunities for enhanced stability, controlled release, and advanced applications in chemical biology.^[^ ^27^, ^28^, ^29^ ^]^

**Figure 1:**
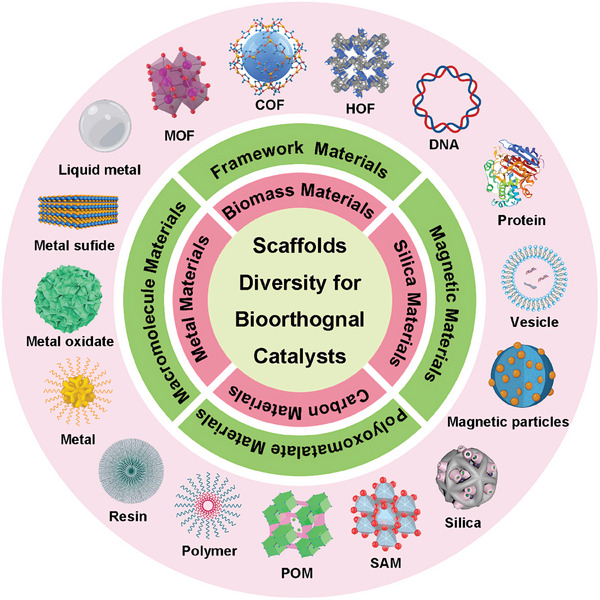
Schematic illustration of the scaffold's diversity for biorthogonal catalysts. The inner circle presents different material categories for bioorthogonal catalysts (magenta and light green region). The outer circle shows various existing examples of nanomaterial scaffolds for bioorthogonal catalysts (pink region).

In the past few years, many researchers have explored new bioorthogonal catalysts based on different scaffolds.^[^ ^27^ ^]^ Although the most catalytic center is still TMCs, the various scaffolds materials play a crucial role in modulating the catalytic activity, stability, and selectivity of the bioorthogonal catalysts, enabling their properties to be tailored to specific biological applications. These new catalysts, with their unique structural and functional features, hold great promise for addressing existing limitations and opening new avenues for bioorthogonal transformations in living things. Moreover, the discovery and exploration of these new catalysts with unique scaffolds have further enhanced the field of bioorthogonal chemistry (**Figure** **1**). In this review, we aim to provide a comprehensive overview of recent advances in bioorthogonal catalyst research based on all potential scaffold designs. We will discuss the design principles, mechanisms of action, reaction rate, and catalytic type of these novel catalysts. We also discuss the features and advantages of different scaffold materials. Furthermore, we will highlight the challenges and future directions in this rapidly evolving field, emphasizing the potential impact of scaffold diversity on advancing bioorthogonal chemistry.

## 2 Diverse Scaffolds of Bioorthogonal Catalysts

TMCs are a class of catalysts that are composed of a transition metal center. They have played a crucial role in many industrial and organic synthesis processes. In the context of bioorthogonal chemistry, TMCs have emerged as powerful tools for bioorthogonal applications due to their ability to catalyze a broad range of reactions with high selectivity and efficiency.^[^ ^22^, ^30^, ^31^ ^]^ However, their direct use within living systems still presents notable challenges. Notably, TMCs usually exhibit poor solubility in aqueous environments, both in vitro and in vivo. More importantly, they are also susceptible to deactivation owing to fluctuations in pH, interactions with serum proteins, and reactions with endogenous molecules (such as thiols) in biological environments. Furthermore, concerns regarding “naked” TMCs include potential acute cytotoxicity, restricted cellular uptake, and challenges associated with their in vivo behavior.^[^ ^32^, ^33^ ^]^

Nanomaterials provide a potential solution to overcome the challenges of TMCs mentioned above.^[^ ^26^, ^34^ ^]^ First, the surface of nanomaterial scaffolds could be modified with hydrophilic or charged groups easily, which enhances the solubility of TMCs by preventing their aggregation or precipitation. This improved solubility allows for efficient dispersion of TMCs in biological or aqueous media.^[^ ^35^ ^]^ Second, nanomaterials offer a protective environment that shields TMCs from external factors such as pH, oxygen, or reactive species. This protection helps preserve the structural integrity and catalytic activity of TMCs, enabling their long‐term stability and functionality.^[^ ^36^ ^]^ Third, by modifying the surface chemistry or introducing specific functional groups, the access of substrates to the catalytic sites of TMCs can be controlled.^[^ ^37^, ^38^, ^39^ ^]^ Besides, Many nanomaterials exhibit excellent biocompatibility, allowing for the safe and effective utilization of TMCs in areas such as in situ drug synthesis, targeted drug delivery, bioimaging, or tissue engineering.^[^ ^40^ ^]^

Therefore, the encapsulation of TMCs into nanomaterials scaffolds generates bioorthogonal “catalysts”, presenting a viable strategy for leveraging TMCs in the field of biomedicine.^[^ ^41^ ^]^ One way to classify bioorthogonal catalysts is based on the kind of scaffolds that are used to incorporate TMCs into the nanomaterials, for instance, polymeric nanoparticles, resin microspheres, metal nanomaterials, proteins, and so on.^[^ ^42^ ^]^ The different scaffolds can provide different properties and functions for the bioorthogonal catalysts, such as solubility, stability, biocompatibility, catalytic activity, and additional functionalities.^[^ ^43^ ^]^ At the end of section 2, a table provides a concise summary of the studies for the new catalysts, including the catalytic center, scaffold materials, and other key details (**Table** **1**)

### 2.1 Polymer Scaffolds

Polymer materials find diverse applications across various fields due to their unique properties and versatility.^[^ ^44^, ^45^, ^46^ ^]^ In this regard, the usage of various formulations comprising biocompatible and degradable polymers emerges as an appealing approach for developing bioorthogonal catalysts. With their wide range of functionalities, polymers provide versatile platforms for the development of “soft” catalysts. Distinguished by their flexible property, these bioorthogonal polymeric catalysts are different from “solid” nanoparticles and coined as “polyzymes”.^[^ ^47^ ^]^ Polymer scaffolds, whether synthetic or natural, can be designed to form hollow nanoparticles, offering several advantages. They protect the TMCs against degradation and leaching, while simultaneously creating a favorable microenvironment for catalytic reactions. Additionally, the versatility of polymer scaffolds enables the incorporation of supplementary functionalities.^[^ ^48^ ^]^ The polymer chains can be modified with targeting ligands, imaging probes, or stimulus‐responsive units, thereby facilitating the development of multifunctional bioorthogonal catalysts. Expanding the range of applications, these systems can be utilized in targeted drug delivery, biosensing, and bioimaging.

**Figure 2:**
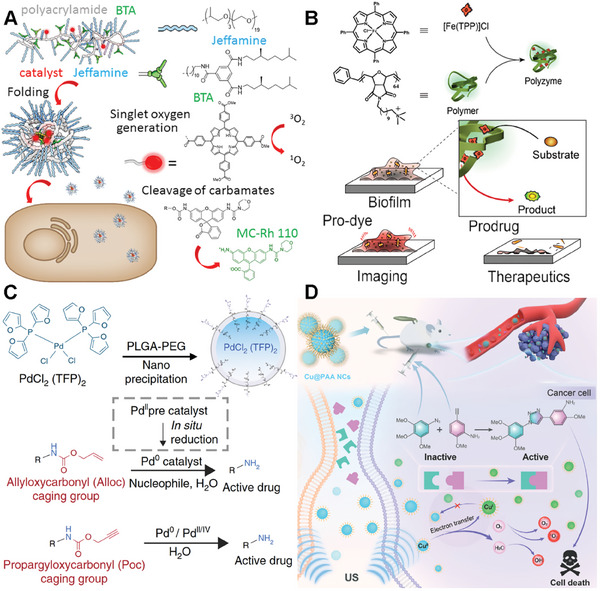
The examples of the bioorthogonal catalysts in polymer scaffold. A) Schematic illustration of these dynamic self‐complementary polymer networks, the single oxygen generation, and cleavage of carbamates. Reproduced with permission.^[^ ^50^ ^]^ Copyright 2013 Wiley‐VCH GmbH. B) Schematic illustration of the polymer‐based catalysts prepared through nanoprecipitation via Fe catalyst and poly(oxanorborneneimides) polymer for imaging and eradication of bacterial biofilms. Reproduced with permission.^[^ ^53^ ^]^ Copyright 2020 American Chemical Society. C) The Pd‐NPs catalyst was designed by encapsulating bis[tri(2‐furyl)phosphine] palladium(II) dichloride within a biocompatible poly(lactic‐co‐glycolic acid)‐b‐polyethylene glycol platform. Reproduced with permission.^[^ ^16^ ^]^ Copyright 2024 Nature Research. D) Representation of Cu@PAA NCs fabrication and US‐controlled site‐specific bioorthogonal catalytic reaction to activate drug and trigger robust sonodynamic therapy. Reproduced with permission.^[^ ^54^ ^]^ Copyright 2023 Wiley‐VCH GmbH.

A typical example of a bioorthogonal catalyst based on polymer scaffolds was developed by the Palmans‘s group with polyacrylamide copolymers.^[^ ^48^, ^49^, ^50^, ^51^, ^52^ ^]^ In this work, the amine ligands in the side chains (bipyridine or phenanthroline) facilitated the encapsulation of TMCs, and the PEG side chains (Jeffamine@M‐1000) were incorporated to address solubility. The folding of these dynamic self‐complementary polymer networks (SCPNs) relied on reversible intramolecular hydrogen bonding in combination with a collapse driven by hydrophobic interactions (BTA) (**Figure** **2**A). The study first provided diverse delivery strategies enabling the selective localization of SCPNs within specific cellular compartments. Notably, the porphyrin‐modified SCPNs in this study generated singlet oxygen successfully under irradiation, inducing localized toxicity. In addition, Pd(II) and Cu(I)‐loaded SCPNs demonstrated successful depropargylation of protected rhodamine in the extracellular environment. These strategies provided promising outcomes, further paving the way for in vivo bioorthogonal catalysis by polyzymes in biomedical applications.

Besides, the Rotello's group successfully synthesized a new polyzyme based on quaternary ammonium polymer (Figure 2B).^[^ ^53^ ^]^ These polymers featuring hydrophobic alkyl side chains self‐assemble into nanoparticles, creating a protective hydrophobic environment for encapsulating TMCs (Fe(TPP)Cl). Furthermore, the cationic feature of this polymer expedites the penetration of these nanoparticles into biofilms. In vitro activation of a non‐fluorescent pro‐dye was conducted using both polyzyme and free catalyst molecules to demonstrate the penetration capability of the polyzyme. Remarkably, bright fluorescence was exhibited in biofilms treated with the polyzyme, while minimal activation was observed in biofilms incubated with free catalyst molecules. The therapeutic potential of the bioorthogonal polyzyme platform was showcased through the activation of a pro‐antibiotic, effectively eradicating bacterial biofilms.

The study by Miller et. al. delves into the novel application of nano‐palladium (Pd‐NP) catalyst for in vivo chemistry based on polymer scaffolds. marking a significant advancement in the field of biomedical research.^[^ ^16^ ^]^ They developed Pd‐NPs by encapsulating bis[tri(2‐furyl)phosphine] palladium(II) dichloride within a biocompatible poly(lactic‐co‐glycolic acid)‐b‐polyethylene glycol platform (Figure 2C). Through meticulous experimentation using mouse models of cancer, the study showcases the efficient accumulation of Pd‐NPs in tumors, where they successfully activated various model prodrugs. This innovative approach not only inhibited tumor growth and extended survival in animal models but also demonstrated reduced toxicity compared to conventional doxorubicin formulations. The findings underscore the safe and effective in vivo catalytic activity of palladium compounds, opening new avenues for targeted and efficient disease treatment strategies.

Chen's group also developed a new biorthogonal Cu core catalyst based on a polymer scaffold. They introduced a novel approach to construct an intelligent bioorthogonal catalyst for cancer therapy.^[^ ^54^ ^]^ In their work, the ultrasmall poly(acrylic acid)‐modified copper nanocomplexes (Cu@PAA NCs) were synthesized via a facile one‐step hydrothermal strategy (Figure 2D). More interestingly, the Cu@PAA NCs exhibit high catalytic efficiency, which can be dynamically modulated through the reversible interconversion of Cu(II) and Cu(I) valence states under ultrasound irradiation. This unique characteristic enables the Cu@PAA NCs to activate prodrugs at the tumor site through a Cu(I)‐catalyzed azide–alkyne cycloaddition reaction, while also generating reactive oxygen species (ROS) for synergistic sonodynamic therapy. The nanocomplexes demonstrate enhanced contrast in both magnetic resonance (MR) and photoacoustic imaging, allowing for multimodal imaging‐guided therapy. Besides, the Cu@PAA NCs are biocompatible, rapidly metabolized, and efficiently eliminated from the body via the urinary system. The study presents a promising strategy for ultrasound control of in situ drug synthesis and therapy, with potential applications in disease theranostics.

### 2.2 Metal‐Related Scaffolds

In a similar vein, Metal scaffolds are metallic or metal‐related nanoparticles that can directly act as TMCs or support TMCs on their surfaces.^[^ ^55^, ^56^, ^57^, ^58^ ^]^ Metal scaffolds offer a unique set of advantages and serve as promising platforms for the development of “rigid” catalysts. Distinct from polymer‐based catalysts, these metal‐based constructs can be referred to as artificial “metallozymes”.^[^ ^59^, ^60^ ^]^ Metal scaffolds, whether inorganic or organometallic, can be engineered to form nanoparticles with well‐defined structures, providing precise control over their catalytic properties. These artificial metallozymes offer exceptional stability against degradation, ensuring their longevity and functionality in complex biological environments. The robustness of metal scaffolds also allows for the incorporation of diverse catalytic centers, enabling a wide range of enzymatic activities and substrate specificities.^[^ ^61^ ^]^ Moreover, metal scaffolds offer the opportunity to anchor additional functionalities in bioorthogonal catalysts. The surface of metal nanoparticles can be functionalized by targeting ligands, imaging agents, or stimuli‐responsive moieties, expanding their capabilities and facilitating multifunctionality. This integration of supplementary functionalities enables the development of metallozymes with enhanced selectivity, improved biodistribution, and the ability to respond to specific cues or stimuli.^[^ ^62^, ^63^ ^]^

#### 2.2.1 Metal Element Materials

**Figure 3:**
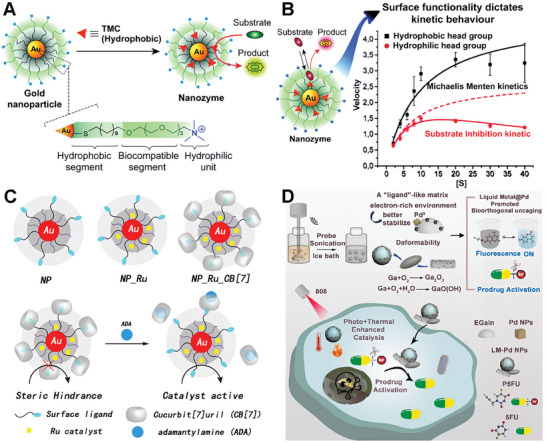
Metal‐related scaffolds for bioorthogonal catalyst designs. A) Gold nanoparticles with appropriate monolayer architecture efficiently encapsulate hydrophobic TMCs to form catalysts. Reproduced with permission.^[^ ^64^ ^]^ Copyright 2018 Wiley‐VCH GmbH. B) Catalysts with different monolayers show different kinetic curves. The hydrophilic surface leads to a kinetic difference from the expected Michaelis–Menten kinetics (dashed line) due to substrate inhibition. Reproduced with permission.^[^ ^66^ ^]^ Copyright 2017 The Royal Society of Chemistry. C) Schematic description of the in situ activation of perfluoro and prodrugs in vivo by Ru catalyst via host–guest interaction. Reproduced with permission.^[^ ^39^ ^]^ Copyright 2024 Elsevier. D) Illustration of the design of liquid metal‐mediated Pd^0^‐based bioorthogonal catalysts and the intracellular prodrug activation by LM‐Pd catalysts. Reproduced with permission.^[^ ^68^ ^]^ Copyright 2023 Elsevier.

One example of a bioorthogonal catalyst on metal scaffolds is one composed of gold element nanoparticles. The Rotello's group synthesized 2–7 nm gold particles via the Brust‐Schiffrin two‐phase synthesis method to create self‐assembled monolayers.^[^ ^64^ ^]^ As shown in **Figure** **3**A, the ligand polymer of the gold nanoparticles usually contains three key components. The hydrophobic segment typically consists of a hydrophobic aliphatic chain with a thiol terminal group, which is attached to the gold core and encapsulates the hydrophobic TMCs. The biocompatible segment typically consists of a hydrophilic ethylene glycol chain, which imparts aqueous solubility and biocompatibility. The hydrophilic unit part usually means a headgroup that can be varied to impart targeting behavior. A number of hydrophobic TMCs (e.g., Pd, Rh, Fe, and Ru) can be embedded into the systems to create various bioorthogonal catalysts performing specific functions in complex biological environments.^[^ ^65^ ^]^ The ingenious design of the ligands ensures stability by protecting and insulating the TMC from external deactivating conditions in the biological environment. Besides, the authors demonstrated the metallozymes could biomimetically control reaction kinetics via varying the ligand structure (Figure 3B).^[^ ^66^ ^]^ This study proved that metallozymes hold immense potential for advancing biomedical research and facilitating the development of innovative therapeutic and diagnostic platforms.

The Rotello's group also cleverly expanded the application of gold‐based bioorthogonal catalysts. They developed protein‐sized bioorthogonal catalysts by encapsulating hydrophobic TMCs within a monolayer of water‐soluble gold nanoparticles with core diameters of ≈2 nm (Figure 3C).^[^ ^67^ ^]^ They selected ruthenium‐catalyzed deallylation as a representative bioorthogonal process for demonstrating the regeneration of fluorescence. The sample TMC[Cp*Ru(cod)Cl] (Cp* = pentamethylcyclopentadienyl, cod = 1,5‐cyclooctadiene) was immobilized in the hydrophobic region of the Au‐polymer layer, resulting in the formation of NP_Ru. The catalytic activity of these systems can be dynamically regulated through the reversible binding of a supramolecular cucurbit^[^ ^7^ ^]^ uril to the monolayer surface, mimicking the allosteric regulation of enzymes (NP_Ru_CB).^[^ ^7^ ^]^ This system allowed for the controlled cleavage of allylcarbamates for pro‐fluorophore activation and propargyl groups for prodrug activation within cellular environments, showcasing its potential for applications in biomedical imaging and therapeutics. The study presents a biomimetic control mechanism for regulating synthetic catalytic systems in cells, which could lead to new avenues for therapeutic applications and integrated biological cellular systems.

Qu's group also presented a novel approach to design a palladium catalyst based on a liquid metal (LM) scaffold to enhance bioorthogonal catalysis for tumor inhibition.^[^ ^68^ ^]^ Liquid metal materials refer to metallic substances that are in a liquid state at room temperature or slightly higher temperatures. Unlike most metals, which are solid at room temperature, liquid metals exhibit unique properties and characteristics that make them intriguing for various applications in fields such as electronics, robotics, energy, and biomedical engineering. Drawing inspiration from the enzymatic concept of ligand‐mediated catalysis, the researchers utilized the electron‐rich properties of gallium indium liquid metal (EGaIn) to stabilize Pd^0^ and facilitated the nucleophilic turnover of π‐allylpalladium species, thereby accelerating the uncaging reaction of precursor molecules (Figure 3D). Additionally, the LM's photothermal properties enhanced tumor cell removal through photo‐enhanced catalysis and photothermal effects. Notably, the study demonstrated the exceptional bioorthogonal catalytic activity of the LM‐Pd catalyst in aqueous environments, achieving a catalytic yield of 99% within 20 min, with a rate ≈60 times faster than that of Pd nanoparticles alone. The research provides a new perspective on the application of LMs in bioorthogonal catalysis and offers a promising strategy for the design of bioinspired bioorthogonal catalysts, potentially broadening the application of LMs in disease treatment.

#### 2.2.2 Metal Oxide Materials

**Figure 4:**
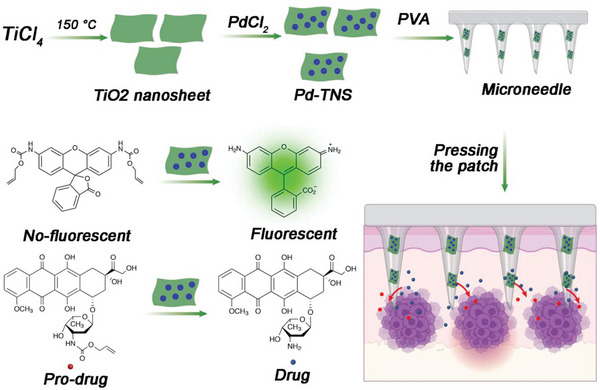
Metal oxides scaffolds for bioorthogonal catalyst designs. a TiO_2_‐based bioorthogonal catalytic device based on a microneedle patch loaded with palladium nanoparticles. Reproduced with permission.^[^ ^69^ ^]^ Copyright 2021 Nature Research.

In addition to metal element scaffolds, some metal complexes can also act as scaffolds for the construction of bioorthogonal catalysts. Gu and coworkers presented a TiO_2_‐based bioorthogonal catalytic device based on a microneedle patch loaded with palladium nanoparticles.^[^ ^69^ ^]^ The device was capable of achieving localized conversion of substrates both inside cells and in vivo (**Figure** **4**). By immobilizing palladium nanoparticles on titanium oxide nanosheet scaffolds, the authors constructed a microneedle patch made of polyvinyl alcohol matrix with high mechanical strength and stability. When the microneedles are placed in a water environment, they swell and form a porous structure, facilitating substrate diffusion and interaction with palladium nanoparticles for activation. The device demonstrated catalytic activity in fluorescent assays and in cell culture experiments, enabling the conversion of prodrugs. Furthermore, in a mouse melanoma model, the microneedle device successfully activated a caged doxorubicin derivative at the tumor site, allowing for increased dosage while limiting side effects on healthy organs and tissues. Overall, this study presents a promising approach for achieving bioorthogonal catalysis both in vitro and in vivo using a microneedle patch, offering potential applications in localized therapy with reduced off‐target effects.

#### 2.2.3 Metal Sulfide Materials

**Figure 5:**
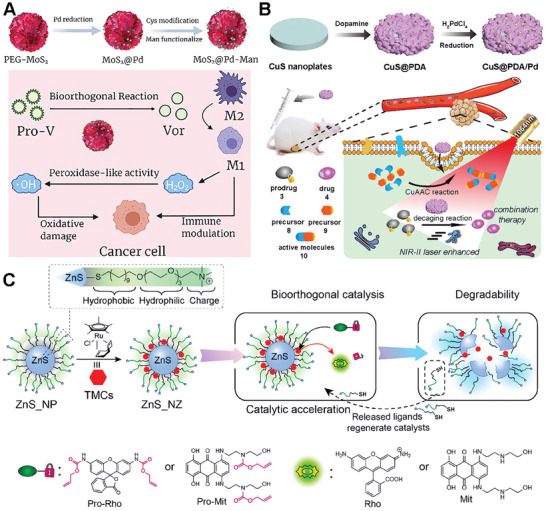
Metal sufide scaffolds for bioorthogonal catalyst designs. A) Schematic illustration of construction and tumor treatment of Pd and mannose decorated MoS_2_ nanoflowers (MoS_2_@Pd‐Man) Reproduced with permission.^[^ ^70^ ^]^ Copyright 2022 Elsevier. B) Schematic presentation of the synthesis of CuS@PDA/Pd and the mechanism of click reaction for in situ dual drug synthesis accelerated by NIR‐II light. Reproduced with permission.^[^ ^71^ ^]^ Copyright 2022 American Chemical Society. C) Schematic presentation of generation of zinc sulfide nanozymes (ZnS_NZ) from zinc sulfide nanoparticles (ZnS_NP). Reproduced with permission.^[^ ^72^ ^]^ Copyright 2022 American Chemical Society.

Similar to metal oxides, metal sulfide materials are also a good class of scaffold materials. Qu and colleagues developed a MoS_2_‐based nanoflower for reprogramming the tumor microenvironment in cancer immunotherapy via bioorthogonal chemistry.^[^ ^70^ ^]^ The MoS_2_ nanoflower was synthesized by a one‐pot hydrothermal synthesis method. Chemical deposition of ultra‐small Pd nanoparticles onto PEG‐MoS_2_ was employed to prepare the bioorthogonal catalyst. (**Figure** **5**A). The bifunctional catalyst, MoS_2_@Pd‐Man, targets M2 macrophages and induces the in situ synthesis of a histone deacetylase inhibitor (vorinostat). The catalyst exhibits peroxidase‐like activity, enhancing tumor synergistic immunotherapy. In in vitro experiments, the catalyst successfully reprogrammed tumor‐associated macrophages (TAMs) to an M1 phenotype and induced the synthesis of vorinostat. In vivo experiments using a colon cancer model demonstrated that the catalyst effectively reprogrammed the TME and significantly inhibited tumor growth. This work presents a novel strategy for cancer immunotherapy by utilizing bioorthogonal chemistry and highlights the potential of bioorthogonal catalysts in reprogramming the TME and improving the efficacy of cancer treatment. This strategy successfully demonstrated synergy therapy effects and highlighted its potential in cancer therapy.

As the biocatalysts are often limited by low reaction efficiency in complex biological environments, much effort has been made to enhance the efficiency. Qu's group presented a novel therapeutic approach for orthotopic combination therapy by leveraging NIR‐II light of Pd‐catalyzed bioorthogonal bond cleavage reactions.^[^ ^71^ ^]^ The authors designed an integrated catalyst, CuS@PDA/Pd (where PDA stands for polydopamine), which was promoted by NIR‐II light to accelerate the reaction efficiency and enable dual bioorthogonal reactions (Figure 5B). The CuS component of the catalyst offers photothermal properties that can be utilized to increase the rate of Pd‐mediated cleavage reactions both in vitro and in vivo, which is particularly beneficial for orthotopic 4T1 tumor treatment. Additionally, CuS catalyzes the synthesis of active resveratrol analogs, a process that, when combined with the Pd‐mediated prodrug activation, results in improved antitumor cytotoxicity. The study demonstrates that NIR‐II light can be effectively used to boost the catalytic activity of transition metals for bioorthogonal combination therapy, providing a potential pathway for more efficient cancer treatments.

The Rotello group has developed a novel platform for bioorthogonal catalysis, utilizing zinc sulfide (ZnS) nanoparticles to support ruthenium catalysts, thereby forming the ZnS_NZ catalyst (Figure 5C).^[^ ^72^ ^]^ The ZnS_NZ nanoparticles exhibit enhanced catalytic activity for the intracellular activation of therapeutics, capable of uncaging alkylated profluorophores and prodrugs. These catalysts are capable of uncaging allylated profluorophores and prodrugs with the ZnS core providing non‐toxicity and degradability. The degradation process of ZnS_NZ results in the release of thiolate surface ligands, which accelerates the rate‐determining step in the ruthenium‐mediated reaction cycles. Compared to non‐degradable gold nanoparticle analogs, ZnS_NZ achieved a ≈2.5‐fold increase in the maximum rate of reaction. The therapeutic potential of these bioorthogonal catalysts is demonstrated by the efficient activation of a chemotherapy drug from an inactive prodrug, resulting in the effective killing of cancer cells. This study highlights the potential of the ZnS catalyst as a biocompatible platform for safe and efficient in situ generation of imaging and therapeutic agents, promoting the clinical translation of bioorthogonal catalysis.

### 2.3 Resin Scaffolds

Resin micro/nanospheres, typically comprised of crosslinked polystyrene, possess a median level of structural integrity, bridging the gap between soft polymers and inorganic materials.^[^ ^73^ ^]^ When exposed to suitable solvents, these resin particles undergo swelling; however, their crosslinked polymer chains prevent rearrangement and preserve the original particle geometry. These scaffolds effectively shield the encapsulated TMCs from the extra conditions, while their porous feature enables precursor molecules to access the catalyst. Resin‐based scaffolds for bioorthogonal catalysts have been successfully created with sizes ranging from nanometers to micrometers.^[^ ^74^ ^]^ These scaffolds exhibit biologically inert properties and minimize cytotoxicity associated with the encapsulated TMCs. One key advantage of resin scaffolds is their structural stability, providing a robust framework for the immobilization of catalytic entities. The interconnected porous structure of resin matrices allows for efficient loading and immobilization of catalytic components, ensuring their retention and activity over extended periods.^[^ ^75^ ^]^ This stability protects the catalytic entities from degradation or leaching, enhancing the longevity and performance of the bioorthogonal catalyst. What's more these resin catalysts preserve their activity when dried or stored for extended durations.

**Figure 6:**
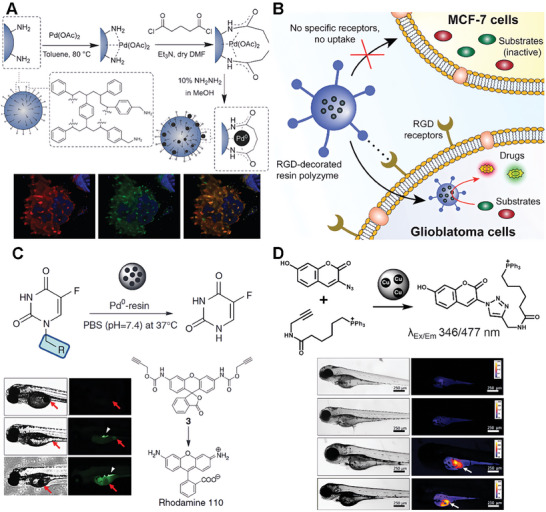
Resin‐based scaffolds for bioorthogonal catalyst designs. A) Preparation of resin microspheres encapsulating Pd nanoparticles. Reproduced with permission.^[^ ^26^ ^]^ Copyright 2021 Royal Society of Chemistry. B) Selective uptake of the resin polyzyme and intracellular activation of anticancer drugs. Reproduced with permission.^[^ ^77^ ^]^ Copyright 2017 Wiley‐VCH GmbH. C) Schematic description of a novel bioorthogonal approach for the activation of prodrugs using extracellular palladium‐catalyzed dealkylation. Reproduced with permission.^[^ ^79^ ^]^ Copyright 2014 Nature Research. D) The work applied extracellular copper catalyst (E‐Cu‐NP) in azide‐alkyne cycloaddition chemistries within biological systems, ranging from cells to zebrafish. Reproduced with permission.^[^ ^80^ ^]^ Copyright 2016 Wiley‐VCH GmbH.

The first Pd^0^‐based heterogeneous catalyst based on resin scaffolds was developed by Bradley's lab. In their work, the amino‐functionalized polystyrene microspheres were synthesized through dispersion polymerization. The study further provides a way to entrap the TMCs in the polymer microspheres after the polymer crosslinking (**Figure** **6**A).^[^ ^76^ ^]^ The authors successfully introduced the Pd^0^ microspheres into HeLa cells and carry out Suzuki cross‐coupling reactions intracellularly, showcasing the potential for cellular synthesis of fluorescent compounds and activated drugs. This work establisheed a foundation for tailoring heterogeneous unnatural catalysts as versatile tools for innovative applications in chemical biology. More importantly, this pioneering study in the field of bioorthogonal catalysts represented a significant milestone. For the first time, the researchers successfully combined transition metal complexes (TMC) with nanomaterials to achieve biocompatible bioorthogonal catalytic reactions. The findings of this study lay a solid foundation and pave the way for further advancements and investigations in the exciting realm of bioorthogonal catalysts. Furthermore, the same group decorated the micro‐spheres with a cyclic RGD peptide to address the challenge of specific cellular targeting of active TMCs.^[^ ^77^ ^]^ The authors effectively showcased the design and application of palladium catalysts, demonstrating their ability to selectively target brain cancer (glioblastoma) cells without compromising their catalytic activity. Notably, these catalysts enable the simultaneous intracellular synthesis of two distinct anticancer agents through different mechanisms, significantly enhancing the therapeutic potential of the drugs. This innovative approach represents a significant advancement in the field of drug activation strategies (Figure 6B).^[^ ^78^ ^]^

As inspired by the works of Bradely groups, Unciti‐Broceta, and co‐workers explored the possibilities of extracellular heterogeneous catalysis in a spatially controllable manner. To realize the activation of prodrugs in the extracellular environment, investigated the potential of a robust heterogeneous catalytic system based on Pd^0^‐functionalized polystyrene resins (Pd^0^‐resins) that surpass the size of human cells.^[^ ^79^ ^]^ The study revealed that the combined treatment of prodrug and catalyst exhibits antiproliferative effects comparable to those of the unmodified drug in colorectal and pancreatic cancer cells. The cytotoxic activity of the system arises from the in situ generation of 5‐fluorouracil, as evidenced by live‐cell imaging and immunoassay studies. Furthermore, the researchers implanted Pd^0^‐resins into the yolk sac of zebrafish embryos, observing excellent biocompatibility and localized catalytic activity. (Figure 6C). In addition to palladium‐based catalysts, Unciti‐Broceta and co‐workers also used the same idea to achieve the synthesis of copper‐based catalysts. The entrapped copper nanoparticle catalyst (E‐Cu‐NP) was synthesized from amino‐functionalized TentalGel resin through s similar synthesis way (Figure 6D). They provided the relatively large size of E‐Cu‐NP (≈160 µm) can be utilized to prevent cell uptake and minimize TMC toxicity.^[^ ^80^ ^]^ This work successfully applied E‐Cu‐NP in azide‐alkyne cycloaddition chemistries within biological systems, spanning from cellular to zebrafish models. This work encompassed a wide range of applications, including fluorophore activation and the in situ generation of activated anticancer agents, opening up numerous exciting avenues for further exploration of copper‐catalyzed azide‐alkyne cycloaddition (CuAAC) reactions. The work further highlighted the potential of these inventions for future applications in bioorthogonal chemistry and therapeutic strategies.

### 2.4 Frameworks Materials Scaffolds

#### 2.4.1 MOF

MOF scaffolds are crystalline materials with a porous structure formed through the self‐assembly of polydentate organic ligands connected by metal nodes.^[^ ^81^, ^82^, ^83^ ^]^ The MOFs possess distinctive micro‐structures, offering appealing traits such as high specific surface area, adjustable porosity, and cavities. Consequently, they find extensive applications in diverse fields, including small‐molecule sensing, gas storage, catalysis, etc.^[^ ^84^, ^85^ ^]^ In recent years, the integration of MOF scaffolds with bioorthogonal catalysts has emerged as a promising strategy for developing advanced catalytic systems with enhanced functionality and biocompatibility.^[^ ^86^ ^]^ Especially, the tunability of MOFs offers versatility in bioorthogonal catalyst design. The metal clusters and organic linkers that constitute MOFs can be carefully selected and modified to tailor the catalytic properties. Additionally, another advantage of using MOF scaffolds for bioorthogonal catalysts is their ability to enhance catalytic performance. The inherent porosity of MOFs enables efficient mass transport of substrates to the active sites of catalysts, leading to improved catalytic efficiency and reaction kinetics.^[^ ^87^ ^]^ Moreover, the tunable pore size and surface chemistry of MOFs allow for the selective encapsulation and protection of catalysts, shielding them from degradation and undesired interactions.^[^ ^88^ ^]^ In particular, significant advancements have been made in harnessing MOFs as catalysts to emulate enzymatic activities.

**Figure 7:**
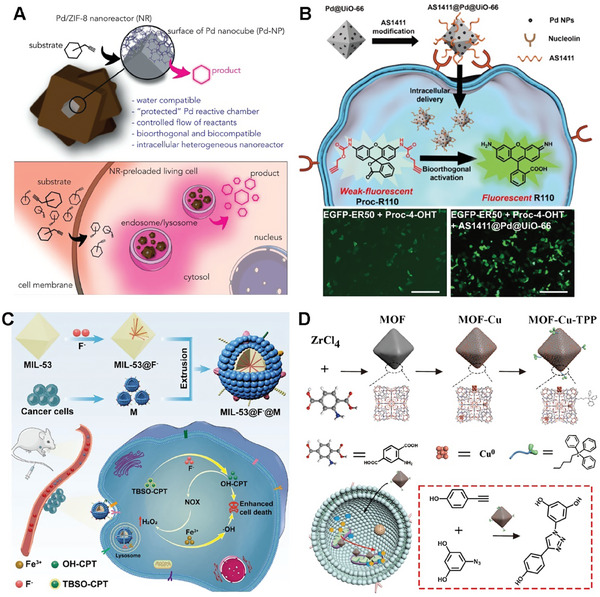
MOF‐based scaffolds for bioorthogonal catalyst designs. A) Scheme for colloidal Pd nanoparticles (Pd‐NPs) coated by a porous ZIF‐8/PMA shell. The NRs tend to accumulate in cytosolic compartments (endosomes/lysosomes) and work as heterogeneous palladium‐based nanoreactors capable of processing substrates, even in a recurrent manner. Reproduced with permission.^[^ ^90^ ^]^ Copyright 2020 Elsevier. B) Schematic illustration of aptamer‐functionalized nanoparticles AS1411@Pd@UiO‐66 for selective bioorthogonal catalysis. The fluorescence image analysis of ER50‐EGFP‐transfected HeLa cells with different AS1411@Pd@UiO‐66 nanoparticles and Proc‐4‐OHT treatment. Reproduced with permission.^[^ ^92^ ^]^ Copyright 2021 American Chemical Society. C) A biomimetic nanocatalyst by camouflaging MOF@F with cancer cell membranes (MIL‐53@F@M), which allows for preferential accumulation in homotypic cancer cells. Reproduced with permission.^[^ ^94^ ^]^ Copyright 2022 American Chemical Society. D) Preparation of the MOF base structure and subsequent decoration with Cu and TPP (MOF‐Cu‐TPP). Activation of resveratrol analog via azide‐alkyne click reaction mediated by the nanoparticle. Reproduced with permission.^[^ ^97^ ^]^ Copyright 2019 Wiley‐VCH GmbH.

Post‐modification of the MOF surface can also overcome common solubility issues in water and biological media.^[^ ^89^ ^]^ In order to develop durable intracellular metallic reactors, Pino et al. presented a water‐compatible core–shell Pd/ZIF‐8 nanocatalyst in living cells and tissue models.^[^ ^90^ ^]^ The research focuses on the design and synthesis of Pd/ZIF‐8 nanocomposites, where Pd nanocubes are selected as active centers due to their shape‐enhanced catalytic performance. The Pd nanocubes are coated with a porous ZIF‐8/PMA shell, providing colloidal and structural stability in water (**Figure** **7**A). The findings of this study highlight the essential role of the MOF‐based shell on Pd/ZIF‐8 in maintaining the integrity of the catalytic chamber, while simultaneously ensuring biorthogonality and biocompatibility. The research also introduces the concept of “catalytic tissues,” demonstrating the potential for recyclable catalytic tissues and the development of ′catalytic cellular or tissue implants. The versatility of nanotechnology promises access to multifunctional nanoreactors in biological settings, with implications for abiotic reactions in living environments. The study provides a broad understanding of the catalytic behavior of the nanoreactors within living cells and tissues, paving the way for future developments in the field of nanoreactors and living tissue models.

Moreover, direct functionalization of MOF surface can endow the MOF catalysts with the ability to actively target cancer cells and the cellular environment.^[^ ^91^ ^]^ For example, to realize the selective bioorthogonal catalysis of the MOF scaffold catalysts, Wang and coworkers constructed a palladium nanocatalyst on UiO‐66 MOF scaffold (Pd@UiO‐66).^[^ ^92^ ^]^ In addition, to enable targeted cancer cell delivery, the Pd@UiO‐66 surface was modified with cancer cell‐targeting aptamer (AS1411).^[^ ^93^ ^]^ AS1411@Pd@UiO‐66 showed a threefold enhancement of catalysis efficiency in cancer cells (Figure 7B). The results were proved by the coincubation with HeLa cells, which exhibited noteworthy enhancements in cellular fluorescence upon exposure to pro‐rhodamine. Besides, the utilization of AS1411@Pd@UiO‐66 nanoparticles for the activation of Proc‐4‐OHT can regulate of stability and activity of EGFP‐ER50 within living cells. This approach, employing cell‐selective bioorthogonal catalysis, offers a robust tool for controlling protein activity and prodrug activation in chemical biology.

In addition to ligand targeting strategies that can achieve cell‐selective targeting of MOF catalysts, biomimetic strategies also provide a good targeting method. Qu's group presented a novel cancer cell‐selective metal‐organic framework (MOF) system for the activation of prodrugs and the enhancement of chemodynamic therapy (CDT).^[^ ^94^ ^]^ They constructed a biomimetic nanocatalyst by camouflaging MOF‐F with cancer cell membranes, which allows for preferential accumulation in homotypic cancer cells (Figure 7C). The pH‐responsive nanocatalyst released fluoride and ferric ions, which activated the prodrug tert‐butyl dimethyl silyl (TBS)‐hydroxycamptothecin through desilylation. After that, the activated drug 10‐hydroxycamptothecin (OH‐CPT) could effectively kill cancer cells. Intriguingly, the activated drug OH‐CPT could increase intracellular H_2_O_2_ by triggering nicotinamide adenine dinucleotide phosphate oxidase, amplifying the Fenton reaction induced by the released iron for synergistic CDT. Both in vitro and in vivo experiments demonstrated the versatility of this fluoride‐activated bioorthogonal catalyst for cancer cell‐selective in situ drug synthesis, potentially accelerating the biomedical applications of bioorthogonal chemistry. The work addresses the challenges of cell selectivity and unitary therapy modality in cancer treatment, offering a promising strategy for targeted and synergistic cancer therapy.

Furthermore, by functionalizing the MOF catalyst, it also becomes possible to actively target specific cell organelles within the intracellular environment.^[^ ^95^, ^96^ ^]^ Qu and colleagues successfully created a highly active and stable mitochondria‐targeted heterogeneous copper catalyst based on a metal‐organic framework, known as MOF‐Cu. (Figure 7D).^[^ ^97^ ^]^ They use UiO‐66(NH_2_) as a scaffold to synthesize MOF‐Cu via solution‐based method. Furthermore, the MOF‐Cu was modified by triphenylphosphonium (MOF‐Cu‐TPP) to anchor the mitochondria targeted ability.^[^ ^98^, ^99^ ^]^ The developed catalyst not only exhibited targeted accumulation within the mitochondria of living cells but also catalyzed the conversion of a prodrug from an inactive to an active state in situ. This was achieved by conjugating two precursor molecules, each featuring alkyne and azide termini, to facilitate the localized synthesis of an active drug within subcellular organelles. In vivo experiments conducted in an animal model revealed that the drug synthesized in vivo, catalyzed by MOF‐Cu, exhibited improved therapeutic efficacy and reduced side effects compared to a pre‐synthesized drug, attributable to its restricted spatial distribution. This new strategy provides a more biocompatible and highly effective approach for localized drug synthesis within subcellular organelles.

#### 2.4.2 COF Material

Covalent Organic Frameworks (COFs) are crystalline porous materials formed through the self‐assembly of organic building blocks connected by covalent bonds.^[^ ^100^, ^101^, ^102^ ^]^ Similar to MOFs, COFs possess unique micro‐structures that provide attractive features such as high specific surface area, adjustable porosity, and cavities. These properties have led to diverse applications of COFs in fields such as small‐molecule sensing, gas storage, catalysis, and more. In recent years, COF materials have also been explored as the scaffold for bioorthogonal catalysts. The tunability of COFs allows for the precise design of bioorthogonal catalysts by carefully selecting and modifying the organic linkers and building blocks that constitute the COF structure. Additionally, COFs offer advantages in terms of catalytic performance enhancement.^[^ ^103^ ^]^ Their inherent porosity facilitates efficient mass transport of substrates to the active sites of catalysts, leading to improved catalytic efficiency and reaction kinetics. Moreover, the tunable pore size and surface chemistry of COFs enable selective encapsulation and protection of catalysts, shielding them from degradation and undesired interactions. Notably, significant progress has been made in utilizing COFs as catalysts to mimic enzymatic activities.

**Figure 8:**
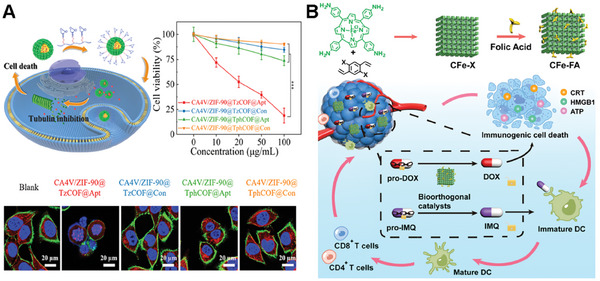
The COF‐based scaffolds for bioorthogonal catalyst designs. A) The general synthesis of CA4V/ZIF‐90@COF@Apt and its mechanism of cancer therapy in DU145 cells. Reproduced with permission.^[^ ^104^ ^]^ Copyright 2022 The Royal Society of Chemistry. B) Illustration of the design and synthesis of COF‐based nanoparticles as a bioorthogonal catalyst and therapeutic mechanism of prodrug activation by CFe−FA nanoparticles. Reproduced with permission.^[^ ^105^ ^]^ Copyright 2023 American Chemical Society.

Song and colleagues introduced a novel bioorthogonal nanoreactor (CA4V/ZIF‐90@TzCOF@Apt) based on the COF/MOF complex‐based scaffolds.^[^ ^104^ ^]^ The click‐activated prodrug, CA4V, was applied for precise anti‐vascular therapy. In their study, CA4V/ZIF‐90 was synthesized by a “one‐pot” self‐assembly strategy (**Figure** **8**A). The bioorthogonal complex catalyst was constructed from the CA4V/ZIF‐90 core and a TzCOF shell. The nanoreactor was modified with aptamer for the enhanced targeted delivery capability. The composite was designed to enhance the click efficiency of CA4V activation and therapeutic effects in vivo. The acid‐induced collapse of the ZIF‐90 core initiates a confined bioorthogonal reaction within the COF cages, which boosts the prodrug's activation and anti‐vascular effects. In vitro and in vivo studies demonstrated that the nanoreactor exhibits targeted delivery and significant antitumor efficacy with minimal off‐target effects, highlighting its potential as a promising strategy for cancer therapy.

The catalyst on pure COF scaffold was presented by Qu and colleagues. They presented a novel approach to construct bioorthogonal catalysts on COF scaffold to realize a bioorthogonal‐activated in situ vaccine for cancer immunotherapy.^[^ ^105^ ^]^ The authors designed a series of biocompatible catalysts to efficiently and safely establish an in situ cancer vaccine (Figure 8B). Specifically, the researchers employed pro‐doxorubicin (pro‐DOX) and pro‐imiquimod (pro‐IMQ) as prodrugs, which are bioorthogonally activated by COF‐based Fe(II) catalysts in situ, resulting in the induction of immunogenic cancer cell death (ICD) and the release of tumor‐associated antigens (TAAs). Moreover, this approach could serve as an adjuvant to amplify antitumor immunity meanwhile. This innovative system not only elicits a potent antitumor immune response but also mitigates dose‐dependent side effects of chemotherapeutic drugs, such as systemic inflammation. The COF‐based bioorthogonal catalysts offer a novel paradigm for the development of personalized cancer vaccines, holding great promise for revolutionizing the safety and efficacy of immunotherapy.

#### 2.4.3 HOF Material

Hydrogen‐bonded organic Frameworks (HOFs) are a class of porous materials composed solely of organic building blocks held together by hydrogen bonds.^[^ ^106^ ^]^ HOFs are constructed through the self‐assembly of organic molecules that possess hydrogen bond donors and acceptors. These building blocks can be designed and synthesized with specific functional groups, such as amides, carboxylic acids, or heterocyclic moieties, capable of forming robust hydrogen‐bonding interactions.^[^ ^107^ ^]^ Unlike other framework materials, Metal‐Organic Frameworks (MOFs) or Covalent Organic Frameworks (COFs), which rely on strong covalent or coordination bonds, HOFs are held together by relatively weaker but highly directional hydrogen bonds. This unique feature endows HOFs with several advantages, such as diverse topologies, adjustable pore sizes, easy surface functionalization, and dynamic behavior.^[^ ^108^ ^]^ While still a relatively new class of materials, HOFs have garnered significant interest in versatile research areas. The cross‐fusion of HOF materials and bio‐orthogonal catalysis further expands new ideas for bioorthogonal catalyst design.^[^ ^109^ ^]^

**Figure 9:**
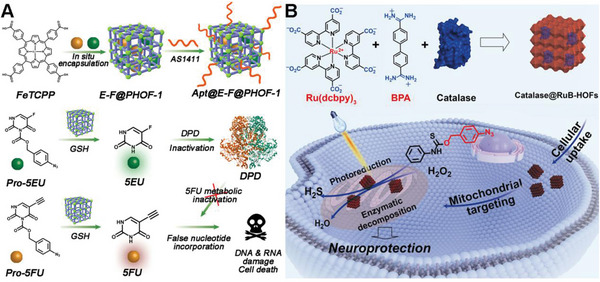
A) Illustration of the synthesis of HOF‐based bioorthogonal catalyst (Apt@E‐F@PHOF‐1) and the chemotherapy mechanism of Apt@E‐F@PHOF‐1 for activation of dual prodrugs. Reproduced with permission.^[^ ^110^ ^]^ Copyright 2022 Nature Research. B) Schematic illustration of the self‐assembly of protein‐encapsulated hydrogen‐bonded organic frameworks (HOFs) to construct the bioorthogonal catalyst (Catalase@Rub‐HOFs) for mitochondria‐targeted bioorthogonal photoreduction and neuroprotection. Reproduced with permission.^[^ ^111^ ^]^ Copyright 2023 Wiley‐VCH GmbH.

Huang et al. presented a cutting‐edge approach to bioorthogonal catalysis aimed at overcoming challenges in drug therapy. The study focused on designing a hydrogen‐bonded organic framework‐based dual prodrugs activation platform, named Apt@E‐F@PHOF‐1 (**Figure** **9**A). This paper aimed to address the critical issues of drug metabolic inactivation and enhancing tumor cell selectivity.^[^ ^110^ ^]^ By utilizing a biocompatible ferric porphyrin HOF‐based bioorthogonal pre‐catalyst, the platform enables the selective activation of 5‐fluorouracil (5FU) and 5‐ethynyluracil within tumor cells. The reduction of ferric porphyrin ligands to ferrous porphyrin by tumor‐specific glutathione triggered the catalytic cleavage reaction, leading to the synthesis of active drugs. Importantly, the presence of the 5‐ethynyluracil inhibitor prevented the metabolic inactivation of 5FU, thereby enhancing the efficacy of chemotherapy while minimizing side effects. Through a combination of in vitro and orthotopic metastatic mouse model experiments, the study demonstrates the potential of this bioorthogonal catalysis platform in improving drug delivery, tumor inhibition, and therapeutic outcomes, highlighting the promise of bioorthogonal chemistry in advancing precision medicine and disease treatment.

Wang's group developed a novel strategy for mitochondria‐targeted bioorthogonal catalysis using protein‐integrated hydrogen‐bonded organic frameworks (HOFs) for neuroprotection.^[^ ^111^ ^]^ The authors successfully synthesized nanoscale RuB‐HOFs (Figure 9B), which exhibited high photocatalytic reduction activity, by self‐assembling a catalytically active ruthenium compound with a specific organic molecule (BPA). These RuB‐HOFs efficiently penetrated cells and preferentially localized to mitochondria, where they facilitated bioorthogonal photoreduction reactions. Notably, RuB‐HOFs encapsulating catalase were found to produce hydrogen sulfide (H_2_S) in mitochondria through photocatalytic reduction of a proH_2_S compound, while simultaneously degrading hydrogen peroxide through enzymatic catalysis, thereby conferring a significant neuroprotective effect against oxidative stress. This study introduces a versatile chemical toolset for mitochondria‐targeted bioorthogonal catalysis, enabling prodrug activation and paving the way for potential therapeutic applications in the treatment of diseases related to cellular oxidative stress.

### 2.5 Carbon‐Based Materials

Carbon‐based materials encompass a diverse class of substances that are primarily composed of carbon atoms.^[^ ^112^ ^]^ These materials exhibit a wide range of properties, making them highly versatile and suitable for various applications.^[^ ^113^, ^114^ ^]^ Carbon‐based materials can be broadly classified into four main categories based on the dimensions of 2D nanomaterials (carbon dots, particulate diamonds, and fullerenes), 1D nanomaterials (carbon nanotubes, carbon fibers and diamond nanorods), 2D nanomaterials (graphene, graphite sheets, and diamond nanopla|‐ nanomaterials (carbon film, carbon sponges and fullerite). Carbon composites are formed by combining carbon with a matrix material, such as polymers or metals, resulting in materials with enhanced strength and stiffness. Carbon‐based materials find applications in fields such as energy storage, electronics, environmental remediation, and biomedicine.^[^ ^115^ ^]^ Their unique combination of properties, including high conductivity, chemical stability, and structural integrity, make them indispensable for advancing technology and addressing various challenges.

**Figure 10:**
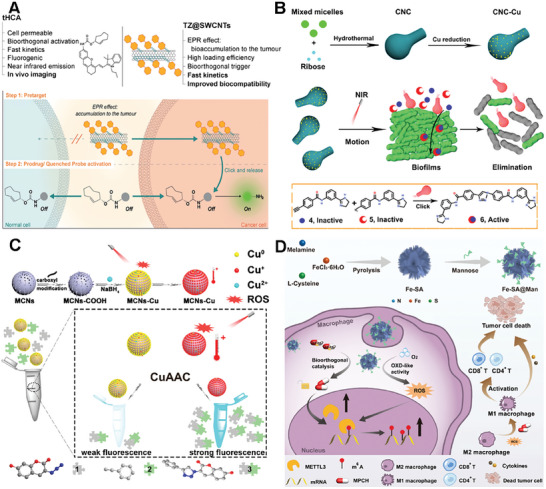
Carbon‐based scaffolds for bioorthogonal catalyst designs. A) Scheme for pretargeted activation strategy using single‐walled carbon nanotubes that bear tetrazines (TZ@SWCNTs). Reproduced with permission.^[^ ^116^ ^]^ Copyright 2020 Wiley‐VCH GmbH. B) Schematic illustration of the fabrication of the carbonaceous nano calabash motor catalyst (CNC‐Cu) and the mechanism of the targeted drug synthesis catalyzed by the CNC‐Cu in the deep‐layered biofilms. Reproduced with permission.^[^ ^117^ ^]^ Copyright 2022 American Chemical Society. C) Illustration of the construction of heterogeneous copper nanocatalyst (MCNs‐Cu) and the mechanism of NIR‐dual‐promoted CuAAC Reaction in living cells. Reproduced with permission.^[^ ^118^ ^]^ Copyright 2020 American Chemical Society. D) Schematic summation of the single‐atom carbon‐based catalysts combined with cancer immunotherapy. The preparation of Fe‐SA@Man NCs and therapeutic mechanism of prodrug activation by Fe‐SA@Man NCs. Reproduced with permission.^[^ ^119^ ^]^ Copyright 2024 American Chemical Society.

Bernardes et al. successfully applied single‐walled carbon nanotubes (SWCNTs) to bioorthogonal chemistry for targeted drug delivery and imaging in cancer treatment.^[^ ^116^ ^]^ The authors employed the bioorthogonal inverse‐electron‐demand Diels‐Alder (IEDDA) reaction between tetrazine and trans‐cyclooctene (TCO) to regulate the release of bioactive agents. In this study, they developed a pretargeted activation approach utilizing single‐walled carbon nanotubes (SWCNTs) modified with tetrazines (TZ@SWCNTs) and a TCO‐caged molecule to deliver active effector molecules (**Figure** **10**A). Notably, they introduced a novel fluorogenic near‐infrared (NIR) probe (tHCA), which can be activated by bioorthogonal chemistry for tumor imaging in mice. Furthermore, this pretargeting strategy enabled the selective activation of doxorubicin prodrug and real‐time tumor visualization with high target‐to‐background ratios in a xenograft mouse model, demonstrating its potential for precise and efficient cancer therapy. The study highlights the potential of functionalized SWCNTs for targeted bioorthogonal approaches with minimal off‐site activation, offering enhanced stability, biocompatibility, and superior pharmacokinetics for cancer therapy and imaging.

Qu's group presented a novel nanomotor catalyst based on a carbonaceous nano calabash (CNC) for deep‐layered bioorthogonal chemistry, which is inspired by self‐propelled biological motors.^[^ ^117^ ^]^ The researchers developed a near‐infrared (NIR) light‐controllable CNC motor catalyst that exhibits good biocompatibility and can actively target the synthesis of drugs within biofilms, a robust platform for bioorthogonal applications (Figure 10B). The CNC motor catalysts, under the influence of a NIR laser, demonstrate rapid autonomous motion and generate active molecules within deep biofilm layers, effectively removing biofilms and eradicating the bacteria they shield. This work provides a new strategy for targeted prodrug activation in bioorthogonal chemistry and has potential implications for treating biofilm‐associated infections and other biomedical applications.

Qu's group also introduced a novel approach to constructing the bioorthogonal catalyst based on mesoporous carbon nanospheres (MCNs) for in vivo applications (Figure 10C).^[^ ^118^ ^]^ They first synthesized highly ordered MCNs via low concentration hydrothermal route and then developed a biocompatible heterogeneous copper nanocatalyst (MCNs‐Cu). Interestingly, the catalytic ability of MCNs‐Cu can be dual‐promoted by NIR light through photodynamic and photothermal effects. The photodynamic activity facilitated the conversion of Cu(0) to Cu(I), accelerating the CuAAC process, while the high photothermal conversion efficiency increases the local temperature, further promoting the reaction. This system demonstrated a significantly increased reaction rate in living systems, from cellular to nematode models, and improved antitumor efficacy in vivo tumor therapy experiments. The study also presents a promising strategy for efficient bioorthogonal catalysis in vivo, with potential applications in targeted drug delivery and cancer therapy.

The Single‐atom catalysts (SACs) based on carbon materials were also explored as the scaffold for bioorthogonal catalysts to overcome the relatively low atomic utilization and low active sites explosion of traditional catalysts. SACs are a class of catalysts where individual metal atoms are dispersed as isolated species on a support material. The unique feature of SACs lies in the presence of single metal atoms as active sites, which offer distinct advantages in catalytic reactions. Single‐atom catalysts have demonstrated remarkable performance in various catalytic reactions, including oxidation, hydrogenation, electrocatalysis, and more. Qu's group presented a novel approach to cancer immunotherapy through the development of sulfur‐doped Fe single‐atom catalysts (Fe‐SA) with atomically dispersed active sites.^[^ ^119^ ^]^ These catalysts, when modified with mannose (Fe‐SA@Man), could selectively regulate the N^6^‐methyladenosine (m^6^A) methylation in macrophages (Figure 10D). The Fe‐SA@Man nanocatalysts activated an agonist prodrug (pro‐MPCH) of the m^6^A writer METTL3/14 complex protein in situ, leading to the upregulation of METTL3/14 expression and hypermethylation of m6A modification. This process reprogramed TAMsto the M1 phenotype, which is essential for effective antitumor immune responses. Additionally, the nanocatalysts exhibited oxidase like activity, further enhancing m6A methylation and macrophage polarization by producing ROS. The study demonstrates the potential of single‐atom catalysts in bioorthogonal catalysis for precise spatiotemporal modulation of cellular processes, offering a new perspective for the treatment of various diseases.

### 2.6 Silica Scaffolds

Silica scaffolds are synthetic materials that are composed of silicon dioxide (SiO_2_) nanoparticles or nanorods.^[^ ^120^ ^]^ Silica is inherently stable and inert, making it an excellent choice for preserving the integrity and activity of catalytic entities. Moreover, Silica scaffolds can be synthesized with various porous structures, such as mesoporous or nanoporous silica, which can be tailored to have different pore sizes, shapes, and surface areas.^[^ ^121^ ^]^ Therefore, silica scaffolds can be designed to encapsulate catalysts within their pores or to conjugate them onto the surface of the silica scaffolds. All of this allows for the creation of silica‐based catalyst constructs with optimized properties, such as enhanced stability, specificity, and substrate affinity. Besides, the silica scaffold can provide a biocompatible and biodegradable environment that can help to reduce the toxicity and immunogenicity of the catalysts.^[^ ^122^, ^123^ ^]^ Owing to the advantages above, Silica scaffolds have emerged as one kind of promising platform for the design of bioorthogonal catalysts.

**Figure 11:**
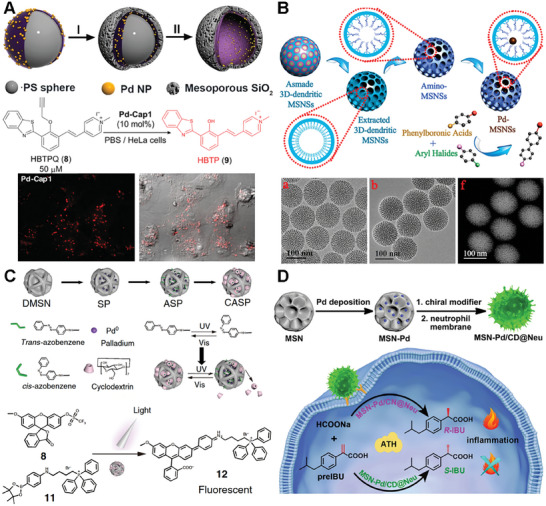
Silica‐based scaffolds for bioorthogonal catalyst designs. A) synthesis of hollow nanoparticles using a polystyrene template and decoration with Pd nanoparticles. Pd‐mediated fluorophore generation (HBTP) in the presence of nanoparticles performed in Vero cells. Reproduced with permission from ref.[13] Copyright 2019 Royal Society of Chemistry. B) Synthesis and catalytic process of highly monodispersed palladium nanoparticles immobilized in th3D dendritic mesoporous silica nanospheres for Suzuki–Miyaura cross‐coupling reaction. Reproduced with permission.^[^ ^127^ ^]^ Copyright 2015 American Chemical Society. C) Schematic illustration of the fabrication of the light‐controlled bioorthogonal catalyst by integrating host–guest interaction and the mechanism of the controllable targeted drug synthesis catalyzed by the CASP in living cells. Reproduced with permission.^[^ ^130^ ^]^ Copyright 2022 Nature Research. D) Fabrication of catalysts by depositing PdNPs into SiNP grooves and covering the particle surface with neutrophil membrane. Targeting of inflammation and Pd‐mediated generation of S‐ibuprofen as the major product. Reproduced with permission.^[^ ^131^ ^]^ Copyright 2020, Elsevier.

One typical example of a bioorthogonal catalyst based on silica scaffolds was developed by Mascareñas and coworkers. They fabricated a hollow mesoporous silica microsphere featuring an inner layer of Pd nanoparticles (**Figure** **11**A).^[^ ^13^ ^]^ In their design, the homogeneous polystyrene beads employed as sacrificial templates were coated with polyallylamine hydrochloride (PAH). Then, freshly prepared Pd nanoparticles were deposited onto these polystyrene particles and subsequently coated with a homogeneous mesoporous silica layer. Afterward, the hollow structure was obtained by removing the polystyrene core via THF. The catalytically active Pd species can uncage propargyl functionalized substrates and facilitate Suzuki–Miyaura cross‐coupling reactions. Thus, catalytic activity of the resulting hollow nanoreactors was detected by uncaging fluorophore HBTPQ in water, PBS, and living cells. To assess the applicability of these Pd‐nanoreactors in more complex bimolecular processes, their biocompatibility and efficiency were also confirmed in epithelial kidney (Vero) cells.

Similarly, in order to address the limitations associated with inefficient mass transport and limited pore accessibility in conventional silica mesostructures, Zhao and coworkers desired colloidal mesoporous nanoparticles with larger pores and a stable framework.^[^ ^124^, ^125^, ^126^ ^]^ They successfully synthesized 3D‐dendritic mesoporous silica nanospheres (3D‐dendritic MSNSs) with a particle size of approximately 125 nm and a pore size of around 6 nm using a biphase stratification approach (Figure 11B). Within the individual mesopore channels of the amino group‐functionalized silica nanospheres, Pd nanoparticles with consistently small size were uniformly dispersed.^[^ ^127^ ^]^ The resulting 3D‐dendritic nanoreactors exhibited high catalytic activity in the Suzuki–Miyaura cross‐coupling reaction. More importantly, the outstanding catalytic performance of these nanoreactors remains remarkably stable, with minimal decrease observed over at least six cycles. The distinctive mesostructure of the 3D‐dendritic MSNSs, characterized by short‐length and large‐diameter mesopore channels, is believed to play a crucial role in effectively immobilizing active and resilient heterogeneous catalysts. This feature holds promising prospects for future catalytic applications.

Compared to the conjugation of TMC nanoparticles in the inner surface of mesopore channels, the of immobilization a TMC to the outer surface of silica nanoparticles can improve the rate of the catalytic efficacy due to the easier substrate access to the center catalyst.^[^ ^128^, ^129^ ^]^ To accomplish this, Qu and colleagues created silica nanoparticles with a grooved surface and embedded Pd nanoparticles within these larger grooves by conjugating them to amine functionalities on the silica surface (Figure 11C). In this work, they developed a light‐controlled bioorthogonal catalyst by integrating a supramolecular complex of azobenzene and β‐cyclodextrin with macroporous silica‐Pd nanoparticles.^[^ ^130^ ^]^ This system allows for the regulation of catalytic activity via light‐induced structural changes, effectively mimicking the allosteric regulation mechanism of biological enzymes. The catalyst demonstrates its utility in cell imaging and the targeted delivery of agents to mitochondria through a Suzuki–Miyaura cross‐coupling reaction, showcasing its potential for precise biochemical research and therapeutic applications. The study's innovative aspect lies in the reversible light‐responsiveness of the catalyst, offering spatial and temporal control over bioorthogonal reactions in living systems.

Furthermore, Qu and colleagues expanded the Pd‐based bioorthogonal catalysis to the field of chiral catalysis.^[^ ^131^ ^]^ In the work, chiral molecule‐modified Pd catalysts were constructed specifically for the purpose of facilitating the asymmetric transfer hydrogenation (ATH) reaction (Figure 11D). Moreover, through the synergistic combination of the ATH reaction and the chemotaxis of neutrophil membranes, they accomplished selective chiral drug synthesis at inflammation sites within living cells successfully. The utilization of neutrophil‐derived cytomembranes, obtained from mouse blood, facilitated the accumulation of nanomaterials specifically at the inflammation site.^[^ ^132^, ^133^ ^]^ This cytomembrane layer enveloped silica‐based catalyst was successfully delivered in vivo, leading to the catalytic uncaging of S‐ibuprofen through the addition of sodium formate as a hydrogen donor. The study showcased the synthesis of chiral drugs through bioorthogonal reactions within living systems, providing a fresh perspective on targeted prodrug activation via bioorthogonal catalysis.

### 2.7 POM Scaffold

Polyoxometalates (POMs) refers to a class of inorganic compounds composed of metal oxide clusters. These compounds are composed of transition metals (typically Mo, W, V, and Nb) in their highest oxidation states and oxide anions.^[^ ^134^, ^135^ ^]^ POMs are highly versatile with a diverse range of compositions and structures, from simple monosubstituted Keggin and Wells‐Dawson structures to more complex Keplerate structures. POMs possess remarkable catalytic capabilities due to their high surface area, redox reactivity, and ability to accommodate guest molecules. They find applications as heterogeneous catalysts in various areas, including materials science, energy conversion, molecular catalysis, and biomimetics. Besides, POMs have shown potential in the field of biomedicine.^[^ ^136^, ^137^ ^]^ They can interact with biomolecules, exhibit antimicrobial activity, and have been explored for drug delivery, imaging agents, and anticancer therapies. Since, POMs have the remarkable ability to undergo reversible multi‐electron redox transformations, making them valuable for modulating the electronic environment of metal nanoparticles in catalysis.^[^ ^138^ ^]^ Due to their unique electronic structures and acid‐responsive self‐assembly capabilities, POMs are considered promising candidates for overcoming the limitations associated with the CuAAC reactions in biomedical applications.

**Figure 12:**
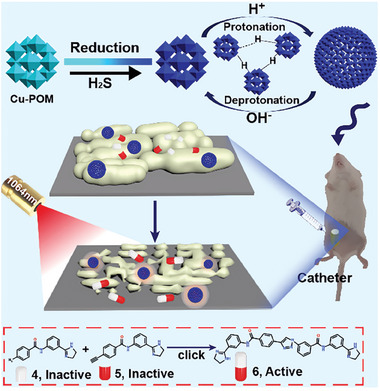
Schematic illustration of the fabrication of the molybdenum (Mo)‐based POM nanoclusters doped with Cu (Cu‐POM NCs) and the mechanism of the selective drug synthesis catalyzed by the Cu‐POM upon laser irradiation for antibiofilm therapy. Reproduced with permission.^[^ ^139^ ^]^ Copyright 023 Wiley‐VCH GmbH.

Qu and coworkers present a novel POM‐based pathologically activated assay for efficient bioorthogonal catalytic selective therapy.^[^ ^139^ ^]^ This work developed a highly efficient bioorthogonal catalyst based on molybdenum (Mo)‐based POM nanoclusters doped with copper (Cu‐POM NCs) (**Figure** **12**). The Cu‐POM NCs are designed to be responsive to pathologically acidic conditions and hydrogen sulfide (H_2_S), which are common in various diseases, for targeted antibiofilm therapy. Leveraging the unique electronic structures and acid‐responsive self‐assembly capabilities of POMs, the Cu‐POM NCs exhibit biofilm‐responsive self‐assembly behavior, efficient CuAAC mediated in situ synthesis of antibacterial molecules, and a NIR‐II (near‐infrared II) photothermal effect selectively triggered by H_2_S in pathogens. The study demonstrates that the Cu‐POM NCs can significantly decrease the number of persister bacteria at the pathological site, which is beneficial for inhibiting bacterial tolerance and eliminating biofilms. The combination of bioorthogonal drug synthesis and photothermal therapy provides an effective strategy for disease‐specific treatment with minimized side effects, offering new insights into the design of efficient and selective bioorthogonal catalysts for disease therapy.

### 2.8 Magnetic Materials

Magnetic materials are substances that possess intrinsic magnetic properties, allowing them to generate and respond to magnetic fields.^[^ ^140^, ^141^, ^142^ ^]^ These magnetic materials, often based on magnetic nanoparticles enable their manipulation and localization in biological systems using external magnetic fields. Magnetic materials find diverse applications in biochemistry, including diagnostics, drug delivery, imaging, and therapy.^[^ ^143^ ^]^ Functionalized magnetic beads and nanoparticles are extensively used for targeted isolation and purification of molecules or cells in techniques such as magnetic separation and immunoassays.^[^ ^144^ ^]^ Magnetic nanoparticles serve as effective contrast agents in MR imaging, allowing for enhanced visualization of specific tissues or molecular targets.^[^ ^145^ ^]^ They also play a role in hyperthermia therapy by selectively heating tumor tissues. Thus, this feature allows for precise positioning of the catalysts at specific sites of interest, enhancing reaction efficiency and selectivity in bioorthogonal chemistry. One of the key advantages of magnetic materials as scaffolds for bioorthogonal catalysts is their ability to facilitate bioorthogonal catalyst immobilization. The magnetic nanoparticles can be functionalized with ligands or linkers that bind to the catalysts, ensuring their stable attachment and preventing leaching into the surrounding environment. This immobilization strategy not only enhances catalyst stability but also enables catalyst recycling, reducing the need for large quantities of expensive catalysts and minimizing waste generation. Moreover, magnetic materials provide spatial control over catalyst localization. By applying external magnetic fields, the magnetic nanoparticles can be directed to specific locations within biological systems, such as cell surfaces or specific tissue regions. This spatial control allows for precise catalyst positioning, leading to enhanced reaction rates and selectivity in bioorthogonal chemistry.

**Figure 13:**
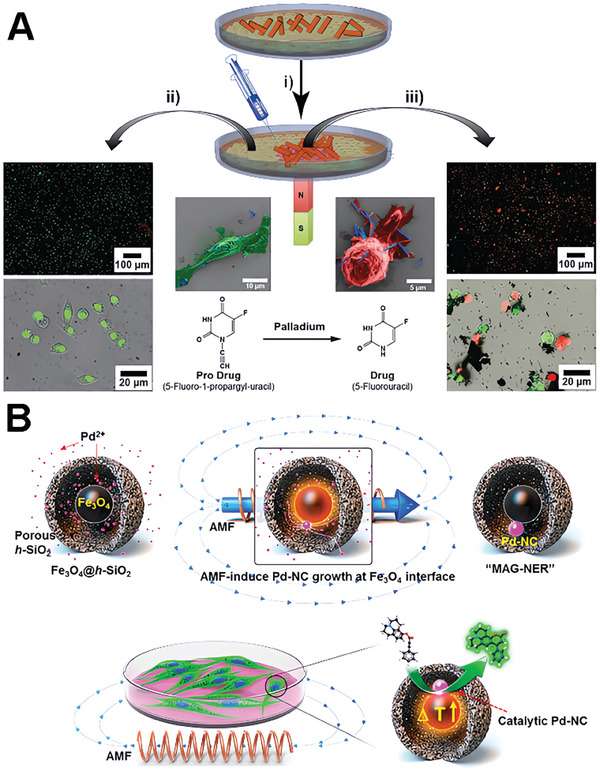
Magnetic‐responsive nanocomposites showcasing on‐demand bioorthogonal catalysis under external stimulation with either static or alternating magnetic fields. A) Magnetic force‐driven accumulation of FePd nanowires for site‐specific prodrug activation. Reproduced with permission.^[^ ^146^ ^]^ Copyright 2022 Wiley‐VCH GmbH. B) Schematics for the synthesis of MAG‐NER and its catalytic applications for AMF‐induced organic catalysis in living cells. Reproduced with permission.^[^ ^147^ ^]^ Copyright 2020 American Chemical Society.

For instance, Hoop et al. introduce a novel approach for targeted cancer therapy using magnetic nanorobots.^[^ ^146^ ^]^ In this study, the fabrication of FePd nanowires relied on template‐assisted electrodeposition within anodized aluminum oxide templates. They showed that FePd nanorobots could accumulate in the regions with a constant magnetic field (CMF), enabling site‐specific bioorthogonal activation of Pro‐5 FU (**Figure** **13**A). The resulting activated drug induces cell death selectively within predetermined cancerous areas. In a proof‐of‐concept experiment, the nanorobots were injected into cancer tumor xenografts, leading to a significant reduction in tumor growth without significant side effects. The study highlights the potential of combining magnetic nanorobotics and bioorthogonal activation of prodrugs as a promising alternative to conventional chemotherapy.

Another strategy entails leveraging the heat‐generating characteristics of magnetic materials when subjected to alternating magnetic fields (AMF) for catalyst activation. Lee et al. present the development of a magnetothermia‐induced catalytic hollow nanoreactor (MAG‐NER) for bioorthogonal organic synthesis in living cells.^[^ ^147^ ^]^ The MAG‐NER is created through the selective growth of palladium nanocrystals on a core–shell structure of iron oxide and silica (Fe_3_O_4_@h‐SiO_2_). These nanoreactors can act as artificial catalytic organelles within living cells, allowing for on‐demand, high‐performance intramolecular ring‐closing reactions (Figure 13B). In the presence of an AMF, the Fe_3_O_4_ component generates heat, enhancing the activity of Pd and expediting bioorthogonal reactions. This approach, which confines local heat generation to the catalytic sites, effectively mitigates macroscopic temperature interference with physiological processes. The use of magnetic fields allows for remotely operated and highly localized heating, making the magnetic materials a promising candidate for bioorthogonal applications.^[^ ^148^ ^]^

### 2.9 Protein Scaffolds

Proteins offer unique properties and serve as highly versatile platforms for the development of “biological” catalysts.^[^ ^149^, ^150^, ^151^ ^]^ Protein scaffolds, derived from natural or engineered proteins, provide a structurally intricate and functional framework for bioorthogonal catalyst synthesis “artificial metalloenzymes”.^[^ ^152^, ^153^ ^]^ One of the key advantages of protein scaffolds lies in their inherent biocompatibility and biodegradability. Proteins are fundamental building blocks of biological systems, and their use as scaffolds ensures compatibility with living organisms. Protein‐based catalysts exhibit excellent biocompatibility, minimizing potential adverse effects and enabling their safe application in various biomedical contexts.^[^ ^154^ ^]^ Moreover, protein scaffolds provide a versatile platform for incorporating additional functionalities and interactions.^[^ ^155^, ^156^ ^]^ Proteins can be modified through genetic engineering or chemical conjugation to introduce targeting ligands, imaging probes, or stimuli‐responsive elements.^[^ ^157^ ^]^ This tunability allows for the creation of protein‐scaffold‐based catalysts with tailored enzymatic properties to suit specific applications.^[^ ^158^, ^159^ ^]^

**Figure 14:**
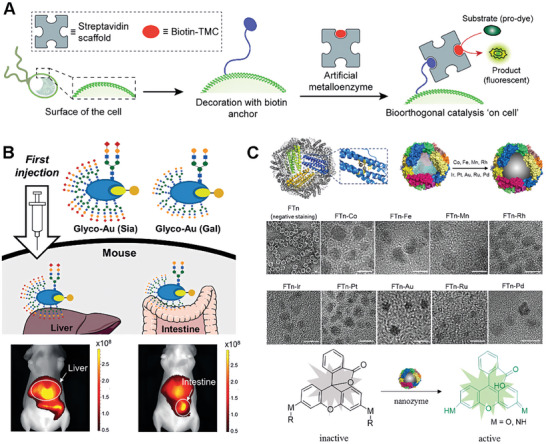
Protein‐based scaffolds for bioorthogonal catalyst designs. A) Modification of the alga cells with the artificial metalloenzyme: both anchor and TMC have a biotin group to bond with streptavidin. Reproduced with permission.^[^ ^160^ ^]^ Copyright 2018 Nature Research. B) Localized catalytic activity using two differently glycosylated albumin for the selective targeting of two different organs: intestine or liver. The catalysis is tracked in vivo by activation of a near‐IR dye. Reproduced with permission.^[^ ^165^ ^]^ Copyright 2017 Wiley‐VCH GmbH. C) Schematic illustration and TEM images (bottom) of various metal‐based catalysts. a catalyst library was established by in situ growth of various metal‐based particles into the FTn inner cavity. Reproduced with permission.^[^ ^166^ ^]^ copyright 2022 the Ivyspring International Publisher.

As a typical example, a biotinylated ruthenium complex was encapsulated within a streptavidin scaffold for the creation of an artificial metalloenzyme.^[^ ^160^ ^]^ To construct the streptavidin‐based TMC, a ruthenium complex ([CpRu(MeCN)_3_]PF_6_) modified biotinylated ligand was tied to the streptavidin scaffold via the biotin‐avidin interaction. This TMC was subsequently anchored onto the surface of an alga cell that had been previously modified with biotin for the next applications. (**Figure** **14**A). The non‐toxic nature to cells of this metalloenzyme was proved by cytotoxicity test. The effectiveness of this approach for bioorthogonal catalysis was further confirmed through the successful uncaging of a coumarin‐based pro‐dye in cells. Moreover, the streptavidin‐biotin catalyst platform has been successfully implemented in mammalian cells using a streptavidin‐based artificial metalloenzyme complexed with a biotinylated cell‐penetrating polymer.^[^ ^161^, ^162^ ^]^ This work utilized cell surface engineering to endue live cells with new‐to‐nature reactivity. In another example, a ruthenium complex was protected using human serum albumin. A ruthenium complex was shielded by human serum albumin to provide protection. The protein was subsequently modified with glycan chains acting as directing ligands, allowing for targeted delivery to cancer cells. This encapsulation of TMCs within a protein scaffold successfully preserved their activity by preventing deactivation caused by endogenous thiols.^[^ ^163^ ^]^ Similarly, Mascareñas and coworkers. Reported catalytically active artificial metalloenzyme inside living mammalian cells by utilizing bis(histidine) miniproteins stapled with palladium(II) complexes.^[^ ^164^ ^]^ They demonstrated that the metallopeptides are efficiently internalized into cells and can promote palladium‐catalyzed depropargylation reactions within the cellular environment. The study highlights the importance of both the peptide scaffold and the palladium staple for achieving intracellular reactivity. These works represent a significant step toward developing bioorthogonal artificial metalloproteins in the native living environment.

Tanaka et al. further present a groundbreaking study on the development and application of a protein‐Au complex for organ‐specific catalysis within live mice (Figure 14B). Their exploration focuses on adapting and utilizing organ‐targeting glycoalbumins as biologically compatible metal carriers to achieve metal complex catalysis.^[^ ^165^ ^]^ The study capitalizes on the strong binding affinity between hydrophobic coumarin and its derivatives with the binding pocket of albumin, enabling the synthesis of the coumarin‐Au conjugate. This study yielded stable Glyco‐Au complexes, specifically a (2‐6)‐disialoglycoalbumin for Glyco‐Au(Sia) and galactosylglycoalbumin for Glyco‐Au(Gal). These complexes were introduced intravenously into live nude mice to investigate their organ‐selective and metal‐catalyzed reactivity. Subsequent injection of fluorescently labeled propargyl ester probes allowed for the visualization of the metal‐catalyzed reactivity within the target organ. The study provides a novel approach for localized propargyl ester amination with nearby proteins within live mice, opening up new possibilities for biomedical and clinical applications.

In addition to targeting specific tissues, Huang et al. studied a protein‐based catalyst platform targeting lysosomes for targeted drug delivery using bioorthogonal catalytic reactions.^[^ ^166^ ^]^ The platform was based on ferritin nanoshells, which were loaded with various transition metals to create metal oxides and metal clusters with catalytic activity (Figure 14C). They found that Pd‐loaded ferritin system had the highest catalytic activity for the cleavage of the propargylic ether bond. They demonstrated that these Pd catalysts could induce lysosomal membrane permeability in vitro and in vivo, leading to the release of the model drug molecule into the cytoplasm. This work proposes that the use of bioorthogonal catalytic reactions to induce lysosomal membrane leakage could be used to deliver a wide range of drugs, including prodrugs, to specific subcellular organelles. It proved the protein‐based scaffold could improve the stability and biocompatibility of the platform and have broad applications in the development of new therapeutic strategies for various diseases via bioorthogonal catalytic reaction.

### 2.10 DNA Scaffolds

The utilization of DNA as a versatile and intelligent building block for the construction of functional nanomaterials has garnered significant attention.^[^ ^167^, ^168^, ^169^ ^]^ The DNA‐templated nano‐materials have been used in various fields, including bioimaging, sensing, and nanoelectronics.^[^ ^170^, ^171^ ^]^ Moreover, the programmability of DNA sequences allows for precise control over the properties and functionalities of scaffolds. By designing specific DNA sequences, it is possible to incorporate catalytic motifs, binding sites, and other functional elements into the DNA scaffold. The design flexibility enables fine‐tuning of the catalyst's catalytic activity, substrate specificity, and overall performance.^[^ ^172^ ^]^ Besides, aptamers, which are single‐stranded DNA or RNA sequences selected through SELEX technology for their high affinity and specificity in recognizing various targets, have found extensive applications in analytical chemistry and nanomedicines.^[^ ^173^ ^]^ By conjugating aptamers onto the surface of nanoparticles, they can selectively bind to overexpressed receptors on tumor cells, and then improve cellular uptake through the receptor‐mediated endocytosis. Owing to the advantages above, the DNA‐based scaffolds have presented as promising approach for the design of bioorthogonal catalysts.^[^ ^174^, ^175^ ^]^

**Figure 15:**
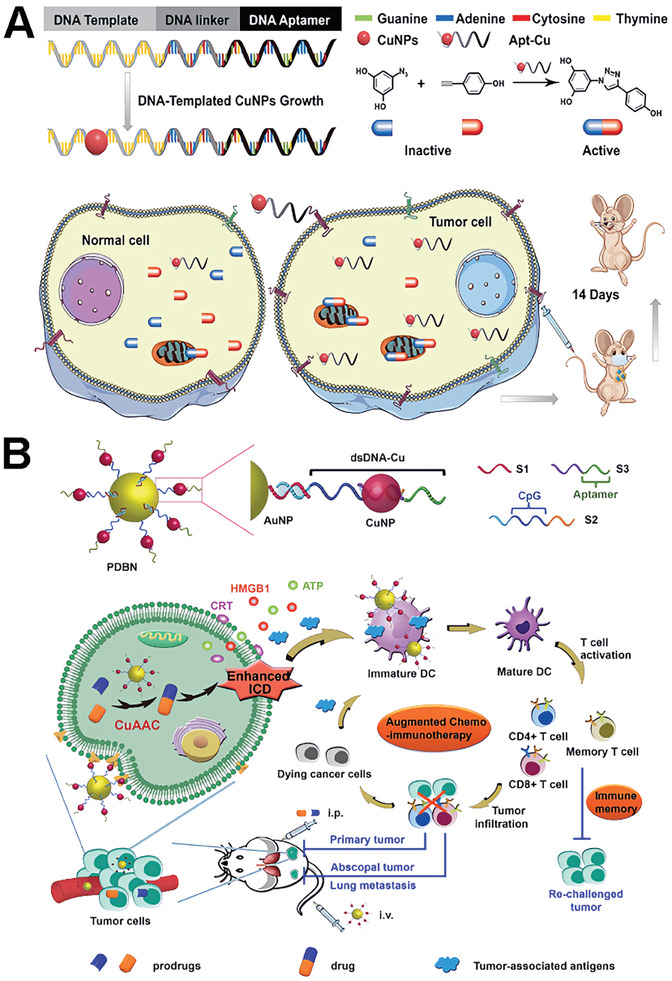
DNA‐based scaffolds for bioorthogonal catalyst designs. A) Illustration of the design and synthesis of DNA‐templated CuNPs as bioorthogonal catalysts. Aptamer‐mediated CuNPs for cell‐specific identification and targeted cancer therapy in vivo. Reproduced with permission.^[^ ^176^ ^]^ Copyright 2022 Nature Research. B) Schematic illustration of the fabrication of the polyvalent DNA‐based bioorthogonal nano agonist and the mechanism of the initiation and augmenting immune responses for robust chemo‐immunotherapy in cancer cells. Reproduced with permission.^[^ ^178^ ^]^ Copyright 2024 Elsevier.

At present, the construction of bioorthogonal catalysts based on DNA scaffolds is still in its infancy, and few studies have utilized this technology. Qu et al. designed and constructed a DNA‐based platform as a biocompatible, highly efficient, and precisely targeted bioorthogonal nanocatalyst.^[^ ^176^ ^]^ The nanocatalyst exhibited remarkable catalytic efficiency in vitro, surpassing the common CuSO_4_/sodium ascorbate catalyst by an order of magnitude (**Figure** **15**A). The theoretical calculation further supported that the superior catalytic activity was owing to the DNA structure and the interaction with substrates. Notably, the system demonstrated remarkable efficiency in activating prodrugs specifically within cancer cells, leading to a 40‐fold increase in transformation compared to non‐targeting catalysts. This enhancement led to improved antitumor efficacy and reduced side effects. Furthermore, in vivo tumor therapy experiments demonstrated the safety and efficacy of this catalyst in mammals. This research will inspire us to combine DNA technology to construct bioorthogonal nanomaterials, which will have broad application prospects in the future.^[^ ^177^ ^]^

Furthermore, Qu's group introduces an innovative approach to enhance chemo‐immunotherapy for cancer treatment. The authors construct a DNA‐based bioorthogonal nano agonist (PDBN) that harnesses the unique properties of nucleic acids to stimulate and enhance immune responses.^[^ ^178^ ^]^ The PDBN, functionalized with gold nanoparticles and copper nanoparticles, serves as an efficient catalyst for the bioorthogonal synthesis of chemotherapeutic drugs in situ, maximizing immunogenic cell death while minimizing systemic toxicity (Figure 15B). As a result, the PDBN triggers the release of tumor antigens and molecular patterns, effectively initiating an antitumor immune response. Furthermore, the PDBN incorporates a high concentration of the immunological adjuvant CpG, which activates antigen‐presenting cells and enhances immune cell activation. The combination of this bioorthogonal catalytic drug synthesis and the immunostimulatory effect of the DNA adjuvant leads to the destruction of both local primary tumors and distant metastases, while also inducing a lasting immune memory effect. This innovative approach expands the application of bioorthogonal chemistry to immunotherapy and offers a safe and potent strategy for cancer treatment.

### 2.11 Extracellular Vesicle Scaffolds

Extracellular vesicles (EVs) refer to small membrane‐bound structures that are released by cells into the extracellular space.^[^ ^179^, ^180^ ^]^ They play essential roles in intercellular communication and are involved in various physiological and pathological processes. According to their biogenesis pathway and size distribution, EVs are further classified into exosomes, microvesicles, and other types.^[^ ^181^ ^]^ Exosomes are a specific subtype of EVs that originate from the inward budding of multivesicular bodies within the endosomal system. These small vesicles, typically ranging in size from 30 to 150 nm, are enclosed by a lipid bilayer membrane exosomes.^[^ ^182^ ^]^ Moreover, exosomes and similar cell‐derived materials exhibit inherent targeting and cell‐specific uptake capabilities.^[^ ^183^ ^]^ These properties arise from the surface proteins and biomolecules present on exosomes, which facilitate their binding and uptake by specific target cells.^[^ ^184^, ^185^ ^]^ Additionally, the small size of exosomes allows for efficient penetration into tissues and cellular uptake, enhancing their potential for therapeutic applications.^[^ ^186^, ^187^, ^188^ ^]^ All the benefits make exosomes an advancing platform for the incorporation and stabilization of bioorthogonal catalysts.

**Figure 16:**
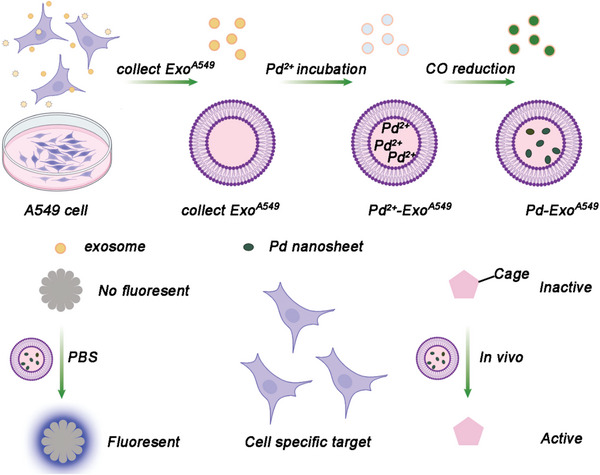
Exosome‐based scaffolds for bioorthogonal catalyst designs. A) Schematic illustration of the preparation process of Pd functionalized exosomes (Pd‐exo^A549^) and the corresponding characterization of the product at each step. B) A confocal study of Pd‐exo^A549^ internalization in A549 cells. The merged confocal images of A549 cells after treatment with Pd‐exoA549 for different hours. C) Pd‐Exo‐mediated conversion of prodrugs to clinically cytotoxic pan‐histone deacetylase (HDAC) inhibitors showed selective action toward A549 cells but not U87 cells. Reproduced with permission.^[^ ^189^ ^]^ Copyright 2019 Nature Research.

Similar to the DNA scaffold above, biologically derived scaffolds deserve further exploration for constructing the bioorthogonal catalysts. As an example, Unciti‐Broceta and colleagues developed a gentle chemical method for the in situ synthesis of Pd nanosheets within exosomes derived from A549 cancer cells (Pd‐Exo^A549^).^[^ ^189^ ^]^ They incubated the exosomes in the supernatant of the A549 cell culture and purified them through multiple cycles of ultracentrifugation. The purified exosome fraction was subsequently suspended in a PBS solution and subjected to treatment with K_2_PdCl_4_, aiming to enhance the internalization of Pd^2+^ ions. Afterward, carbon monoxide (CO) was utilized as a gaseous reductant to synthesize Pd‐Exo^A549^. To assess the catalytic activity of Pd encapsulated within exosomes, the Pd‐Exo^A549^ were incubated with a non‐fluorescent compound (**Figure** **16**A). Through quantitative analysis of the red fluorescent signal, it was confirmed that the maximum cytoplasmic presence of Pd‐loaded vesicles was achieved after incubating cells with Pd‐Exo^A549^ for 6 h (Figure 16B). Furthermore, to explore the homologous targeting capability and catalytic activity of Pd‐Exo^A549^ within the intracellular environment, the researchers conducted cell‐based assays. These assays verified the Pd‐Exo^A549^ could specifically target its parent A549 cells and promote the uncaging reaction within the targeted cell (Figure 16C). This proof‐of‐concept study convincingly demonstrates the potential of bioorthogonal catalysts derived from cancer‐derived exosomes for cancer treatment. Moreover, it opens up new avenues for exosome‐mediated targeted delivery of TMCs, significantly improving the biocompatibility of TMCs and reducing the dose‐dependent toxicity of anticancer drugs.

**Table 1:** Summary of the bioorthogonal catalysts based on different scaffolds.

Scaffolds	Materials	Catalytic center	Catalytic model	Size and Features	Ref.
polymer	poly(oxanorborneneimides), cationic tetra‐alkylamines	Fe(TPP)Cl	homogenous	50 nm size, water solubility, pro‐dye and pro‐antibiotic activation, eradicating bacterial biofilm	[53]
	poly(lactic‐co‐glycolic acid)‐b‐polyethylene glycol platform	PdCl_2_(TFP)_2_	homogenous	60 nm, FDA approved amphiphilic block copolymer, enhanced permeability and retention (EPR) effect, reduced toxicity, cancer therapy	[16]
	poly(oxanorborneneimides)	Cp*Ru(cod)Cl	heterogenous	80 nm, no acute cytotoxicity, in situ activation of pro‐dyes and prodrugs, catalytic centers protection, cancer therapy	[47]
	polyacrylamide	Pd/Cu	homogenous	5‐20 nm, amphiphilic block copolymer, selective localization, universal strategy, singlet oxygen generation,	[40]
	poly(trimethyldecan‐1‐aminium acrylamide)‐co‐poly(azidopropyl acrylamide)	Ru(bpy)_3_	homogenous	10 nm, water‐soluble, folding and crosslinking strategy, intracellular dual catalysis tandem reaction in E.coli., artificial organelles,	[190]
	aspartate‐containing polyolefins	Cu	homogenous	10 nm, highly efficient, low toxicity, low metal loadings, biocompatible click chemistry, antimicrobial drug synthesis	[52]
	polynorbornene	Cu	heterogenous	20‐200 nm, membrane‐embedded catalyst, reduce catalyst poisoning, facilitating the uptake, substrate‐selective in living system	[191]
	polyacrylamide	[CpRu(MeCN_)3_]PF_6_	homogenous	10 nm, neutral polymer, covalent crosslinking free, tandem catalysis, double gated system release	[192]
	poly(acrylic acid)	Cu	homogenous	3 nm, high catalytic efficiency, ultrasound irradiation responsive, magnetic resonance and photoacoustic imaging, synergistic sonodynamic therapy	[54]
Metal element	Gold particles	Cp*Ru(cod)Cl	homogenous	2 nm, enzyme mimic, allosteric regulation, prodrug activation, pro‐fluorophore activation	[67]
	Gold particles	Cp*Ru(cod)Cl	homogenous	2 nm, pH‐switchable, enhanced ability of penetration, biofilms imaging, antibacterial	[193]
	Gold particles	Fe‐TPPCl	homogenous	2 nm, Mannose‐functionalized, selective uptake on macrophages, treating intracellular infection	[194]
	Gold particles	Fe‐TPPCl	homogenous	2nm, reversible thermoresponsive nanocatalysts, reversibly switch, deprotection of Antimicrobials.	[195]
	Gold nanorods	Pd	heterogenous	108 nm, dual‐functional catalyst, in situ prodrug activation, CDT, powerful photothermal agents	[196]
	Liquid metal particles	Pd	heterogenous	210 nm, Enhanced bioorthogonal catalysis, tumor inhibition, photothermal effects	[68]
Metal oxide materials	TiO_2_/Ti‐microdisks	Pd	heterogenous	40–200 µm, local therapy strategy, drug‐eluting devices surgically implantable and detectable	[197]
	titanium oxide nanosheet	Pd	heterogenous	200 nm, microneedle device, high mechanical strength, and stability, limiting side effects	[69]
Metal sulfide materials	MoS_2_ nanoflower	Pd	heterogenous	140 nm, macrophages targeting, Reprogramming the tumor microenvironment, synergistic immunotherapy	[70]
	CuS nanoplates	Pd	heterogenous	20 nm, IR‐II light enhanced, dual bioorthogonal catalysis, combination therapy	[71]
	ZnS	Cp*Ru(cod)Cl	heterogenous	15 nm, enhanced catalytic activity, non‐toxicity, degradability, chemotherapy drug activation	[72]
Resin	polystyrene microspheres	Pd	heterogenous	500 nm, first Pd^0^‐based heterogeneous catalyst, cross‐coupling reaction, allylcarbamate cleavage reaction, intracellular chemistry	[76]
	polystyrene microspheres	Pd	heterogenous	207 nm, RGD peptide modified, glioblastoma cells targeting, two anticancer drugs generation	[77]
	polystyrene microspheres	Pd	heterogenous	150 µm, spatially controllable, zebrafish embryos, minimize TMC toxicity	[198]
	polystyrene microspheres	Cu	heterogenous	160 µm, prevent cell endocytosis, minimize TMC toxicity, CuAAC chemistry, zebrafish models	[80]
	Polystyrene microspheres	Au	heterogenous	75 µm, gold catalysis, locally controlled release, activation in brain, zebrafish models	[15]
	PLGA spheres	AuPd	heterogenous	100 nm, superior catalytic properties, Overcoming Intracellular Deactivation, enhanced transcellular	[199]
MOF	ZIF‐8	Pd	heterogenous	250 nm, core–shell platform, water‐compatible, shape‐enhanced catalytic performance, flow controlled, biocompatibility,	[90]
	UiO‐66	Pd	heterogenous	300 nm, cell‐targeting aptamer, cell‐selective bioorthogonal catalysis, Protein activity regulation	[92]
	MIL‐53	Fe/F‐	heterogenous	120 nm, cancer cell‐selective, cancer cell membranes coating, pH‐responsive release ability, precise cancer therapy	[94]
	UiO‐66	Cu	heterogenous	120 nm, mitochondrial targeting, resveratrol‐derived drugs, greater antitumor efficacy, minimized side effects	[97]
	MOF‐199	Cu	heterogenous	80 nm, cancer cell‐activated, photosensitizers generator, mitochondrial targeting, zebrafish model	[200]
COF	ZIF‐90@TzCOF	IEDDA	heterogenous	63 nm, COF/MOF complex, click‐activated prodrug, enhanced targeted delivery	[104]
	COF‐TAPP‐Dha	Fe	heterogenous	80 nm, in situ vaccine, tumor‐associated antigens, amplify the antitumor immunity	[105]
HOF	Ferric‐porphyrin HOF	Fe	heterogenous	300 nm, aptamer AS1411 modification, dual prodrugs activation, prevention of drug inactivation, enhancing tumor inhibition	[110]
	RuB‐HOFs	Ru	heterogenous	400‐600 nm, mitochondria‐targeted, high photocatalytic reduction activity, hydrogen sulfide production, neuroprotection	[111]
Carbon materials	single‐walled carbon nanotubes	IEDDA	heterogenous	100–300 nm, fluorogenic near‐infrared (NIR) probe production, real‐time tumor visualization, minimal off‐site activation	[116]
	carbonaceous nano calabash	Cu	heterogenous	500 nm, nanomotor catalyst, NIR light‐controllable, deeper penetration depths, removing the biofilms	[117]
	mesoporous carbon nanospheres	Cu	heterogenous	51 nm, biocompatible heterogeneous nanocatalyst, NIR light responsive, dual‐promote the CuAAC reaction, cancer therapy	[118]
	Fe single‐atom catalysts	Fe	heterogenous	100 nm, oxidase like activity, selectively regulate the N6‐methyladenosine (m6A) methylation in macrophages, precise spatiotemporal modulation, cancer immunotherapy	[119]
Silica	hollow mesoporous silica microsphere	Pd	heterogenous	500 nm, hollow microspheres, depropargylation reaction, Suzuki–Miyaura cross‐coupling	[201]
	3D‐dendritic mesoporous silica nanospheres	Pd	heterogenous	125 nm, ultradispersed palladium nanoparticles, stable catalytic activity, excellent performance	[127]
	macroporous silica nanosphere	Pd	heterogenous	80 nm, host–guest interaction, light responsive, allosteric regulation mechanism, targeted delivery of agents	[130]
	macroporous silica nanosphere	Pd	heterogenous	98 nm, chiral molecule‐modified, asymmetric transfer hydrogenation reaction, chemotaxis of neutrophil membranes, chiral drug synthesis	[131]
POM	molybdenum (Mo)‐based POM nanoclusters	Cu	heterogenous	220 nm, in situ drug synthesis, pathologically acid, and H_2_S dual responsive, antibiofilm therapy, NIR‐II photothermal property	[139]
Magnetic materials	FePd nanowires	Pd	heterogenous	5 µm, mobile nanocatalysts, magnetic responsive, prodrug activation, site‐specific bioorthogonal activation	[146]
	iron oxide and silica nanosphere	Pd	heterogenous	42 nm, magnetothermia‐induced nanoreactor, high‐performance, remote operation	[147]
Protein	streptavidin	[CpRu(MeCN)_3_]PF_6_	homogenous	≈8 nm (53 kDa), streptavidin scaffold, cell surface engineering, pro‐dye, and pro‐drug activation	[160]
	human serum albumin	coumarin–ruthenium catalysts	homogenous	≈8 nm (66.5 kDa), artificial metalloenzyme, high enzyme activity, protection from metabolites, anticancer targeting	[163]
	glycoalbumins	Au	homogenous	Over 8 nm (≈98 kDa), stable Glyco‐Au complexes, organ‐specific catalysis, nearby proteins modification	[165]
	ferritin nanoshells	Pd	homogenous	1.1–2.4 nm (42 kDa), mimic mutant P450_BM3_, lysosomal membrane leakage, anti‐cancer therapy	[166]
DNA	double‐strand DNA	Cu	homogenous	1.78–4.79 nm, biocompatible, highly efficient, precisely targeted, prodrugs activation, cancer therapy	[176]
	double‐strand DNA	Cu	homogenous	3.5 nm, DNAzyme‐CuNPs, excellent anti‐cancer effects, minimal side effects, enhance chemo‐immunotherapy	[178]
Extracellular vesicles	A549 exosome	Pd	heterogenous	100–140 nm, cancer‐derived exosomes, homologous targeting capability, uncaging reaction, cancer therapy	[189]

## 3 Conclusion and Future Perspectives

In the realm of chemical biology, the emergence of new bioorthogonal catalysts has opened a plethora of opportunities for the development of innovative therapeutic strategies and diagnostic tools. By leveraging the unique properties of scaffold diversity in catalysis, these catalysts have shown the potential to engage with complex biological systems in a highly specific and controlled manner. This review has highlighted the remarkable progress in designing catalysts that are not only highly efficient and specific but also compatible with the intricate environment of living systems. The versatility of these bioorthogonal catalysts stems from their ability to be tailored from a variety of materials, each offering unique properties and reactivity profiles. This has paved the way for a more nuanced understanding of biological processes and has opened new avenues for the development of targeted therapeutics and advanced diagnostic techniques.

Looking ahead, we also believe that the future of bioorthogonal catalysts is promising. The explosion of different bioorthogonal catalysts on scaffolds offers exciting prospects and opportunities for further exploration and advancement. Several key areas warrant attention to unlock the full potential of these bioorthogonal catalysts:

1. Catalyst Efficiency and Selectivity: Continued efforts should be directed toward improving the efficiency and selectivity of bioorthogonal catalysts.^[^ ^202^ ^]^ This includes understanding the factors that influence catalytic activity, developing strategies for catalyst optimization, and exploring new scaffold designs that enhance reactivity and specificity. By fine‐tuning the catalysts, researchers can expand the repertoire of bioorthogonal reactions and enable precise manipulation of complex biological systems.
2. Biocompatibility and Stability: As bioorthogonal catalysts find applications in biomedical settings, ensuring their biocompatibility and stability is of utmost importance. Similar to the application of most nanomaterials, future research should focus on developing catalysts that are non‐toxic, stable, and compatible with biological environments.^[^ ^203^, ^204^, ^205^ ^]^ This involves considering factors such as cytotoxicity, immunogenicity, and long‐term stability to enable their safe use in vivo.
3. Imaging and Visualization: Integration of bioorthogonal catalysts with imaging modalities is a promising avenue for real‐time visualization and monitoring of biological processes.^[^ ^206^, ^207^ ^]^ Further research should explore the development of catalysts that can be directly visualized using imaging techniques, enabling precise tracking of catalytic activity in living systems. This will provide valuable insights into reaction kinetics, spatial distribution, and overall efficacy.
4. Multifunctionality and Integration: The future of bioorthogonal catalysts lies in their integration with other functionalities and technologies.^[^ ^208^, ^209^, ^210^ ^]^ By combining catalysts with targeting ligands, stimuli‐responsive elements, or drug delivery systems, researchers can create multifunctional platforms that enable site‐specific reactions, controlled release, and personalized medicine. Exploring synergistic interactions between bioorthogonal catalysts and other emerging fields, such as nanotechnology and synthetic biology, will open up new avenues for innovative applications.
5. Clinic Translation and Practicality: Bridging the gap between academic research and industrial applications is vital for the widespread adoption of bioorthogonal catalysts.^[^ ^211^ ^]^ Efforts should be made to optimize catalyst synthesis, scale up production, and develop robust catalytic systems that can be readily employed in practical settings. Collaboration between academia, industry, and regulatory bodies will be essential to ensure the successful translation of bioorthogonal catalysis into commercial products and therapies.

In conclusion, the exploration of new bioorthogonal catalysts with scaffold diversity is a rapidly evolving field with immense potential for revolutionizing chemical biology. The utilization of diverse scaffold types offers exciting opportunities for precise control, spatial manipulation, and multifunctionality in bioorthogonal reactions. Continued research and innovation in this area will drive the development of catalysts with enhanced properties and expand the applications of bioorthogonal chemistry in various biomedical fields, ultimately contributing to advancements in targeted therapy, diagnostics, and imaging.

## Conflict of Interest

The authors declare no conflict of interest.
